# Dendritic ZSM-5 zeolites as highly active catalysts for the valorization of monoterpene epoxides†

**DOI:** 10.1039/d4gc04003a

**Published:** 2024-09-16

**Authors:** Luis A. Gallego-Villada, Jennifer Cueto, María del Mar Alonso-Doncel, Päivi Mäki-Arvela, Edwin A. Alarcón, David P. Serrano, Dmitry Yu. Murzin

**Affiliations:** a Laboratory of Industrial Chemistry and Reaction Engineering, Johan Gadolin Process Chemistry Centre, Åbo Akademi University Henriksgatan 2 20500 Turku/Åbo Finland dmitry.murzin@abo.fi; b Environmental Catalysis Research Group, Chemical Engineering Faculty, Universidad de Antioquia Medellín Colombia alfonso.gallego@udea.edu.co; c Thermochemical Processes Unit, IMDEA Energy Institute Avda. Ramón de la Sagra 3 28935 Móstoles Madrid Spain david.serrano@imdea.org; d Chemical and Environmental Engineering Group, Rey Juan Carlos University c/Tulipán s/n 28933 Móstoles Madrid Spain

## Abstract

Dendritic ZSM-5 zeolites were investigated in the isomerization of monoterpene epoxides, including limonene-1,2-epoxide (LE), α-pinene epoxide, and β-pinene epoxide, which yields high-value compounds used in fragrances, cosmetics, and pharmaceuticals. The fresh catalysts were thoroughly characterized using XRD, Ar physisorption, pyridine-FTIR, TEM, FTIR/DTBPyr, and ^27^Al MAS NMR. In comparison with conventional and hierarchical ZSM-5 materials, the dendritic zeolite with a crystallization time of 4 days (d-ZSM-5/4d) was the most active material, with a turnover frequency value of 4.4 min^−1^ for LE isomerization. Likewise, remarkable yields of dihydrocarvone (DHC, 63%, 70 °C, 2 h), campholenic aldehyde (72.4%, 70 °C, 5 min), and myrtanal (47.7%, 50 °C, 5 min) were obtained with this material that exhibited the largest mesopore/external surface area (360 m^2^ g^−1^), showing also the narrowest mesopore size distribution. A direct relationship was observed between the TOF values and the concentration of external Brønsted acid sites, showing the presence of strong steric/diffusional limitations that are greatly overcome with the dendritic zeolites. The lower reactivity of *trans*-LE compared to *cis*-LE was attributed to the larger steric hindrance of the oxygen atom. Exploration of the solvent influence revealed that the reaction rate of LE was favored by non-polar solvents, while highly selective DHC formation occurred in the solvents of medium polarity. The d-ZSM-5/4d sample was shown to be robust because catalytic activity could be completely recovered by air calcination.

## Abstract 2

Dendritic ZSM-5 zeolites were studied for isomerizing monoterpene epoxides like limonene, α-pinene, and β-pinene epoxides, producing valuable compounds for fragrances, cosmetics, and pharmaceuticals.

## Graphical abstract

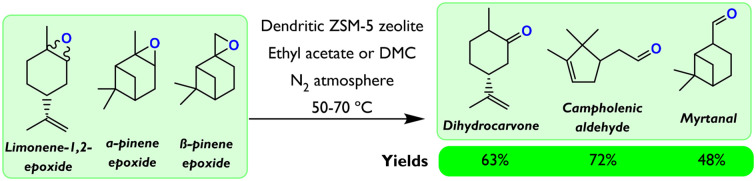

## 1. Introduction

**Fig. 1:**
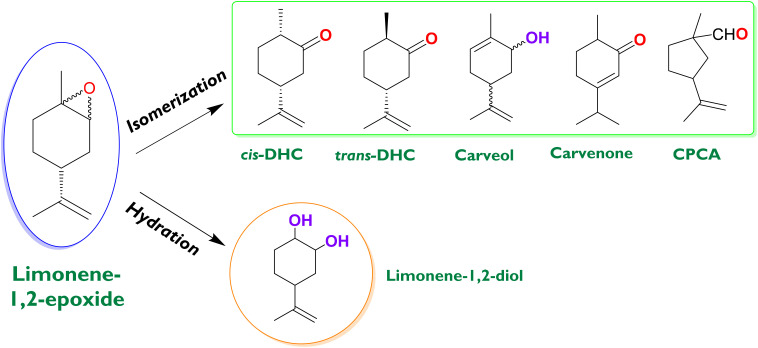
Transformation of limonene-1,2-epoxide into *cis*-dihydrocarvone (*cis*-DHC), *trans*-dihydrocarvone (*trans*-DHC), carveol, carvenone, cyclopentyl-carboxaldehyde (CPCA), and (1*S*,2*S*,4*R*)-limonene-1,2-diol.

The valorization of renewable feedstock has particularly attracted a lot of attention as one of the main areas of chemistry for the production of a wide range of valuable chemicals,^1–5^ always searching for greener systems to contribute to the twelve principles of Green Chemistry.^6^ Among various feedstock, terpenoids constitute one of the most numerous and structurally diverse natural products, which can be extracted by distillation such as α- and β-pinene from turpentine oil, but also utilizing mechanical treatment such as limonene from the peels of citrus fruits.^7–9^ Selective partial oxidation of these platform molecules has been widely investigated through two well-known routes: allylic oxidation often involving free-radical pathways,^10,11^ and the epoxidation of the double carbon–carbon bond to obtain the corresponding cyclic ether.^12–19^ Synthesis of monoterpene epoxides like limonene-1,2-epoxide (LE) presents an enormous significance because these are intermediates for the production of bio-based polymers^20–22^ or can be transformed, usually by isomerization (Fig. 1), into a series of important chemicals used in fragrances, cosmetics, and pharmaceuticals such as dihydrocarvone (DHC) and carveol.^23,24^ DHC is a monoterpenoid compound that can be naturally found in caraway oil, being a key building block to synthesize sesquiterpenes.^25,26^ It can be used in the synthesis of (i) dispiro 1,2,4,5-tetraoxanes, which show potent anti-malarial activity,^27^ (ii) an epoxylactone, which can undergo copolymerization with ε-caprolactone to form cross-linked copolymers with shape memory properties,^26^ and (iii) α-cyperone, which is a eudesmane type sesquiterpenoid compound with potent insecticidal activity.^28^

There is considerable recent literature on the development of efficient heterogeneous catalytic systems for the isomerization of α-pinene^29–38^ and β-pinene^39,40^ epoxides, including also some homogeneous ones.^41–44^ These studies have been mainly focused on the production of campholenic aldehyde and myrtanal, respectively. However, there is a noticeable research gap regarding selective heterogeneous catalysts for the synthesis of DHC *via* the isomerization of internal limonene epoxide (LE). This route (Fig. 1) involves the intramolecular rearrangement of the epoxy group, yielding thermodynamically stable isomers with distinct structures and properties. Specifically, DHC is formed by a hydride shift, whereas other products are generated by alkyl migration.^45^ The major homogeneous catalyst reported for the isomerization of LE is ZnBr_2_, with the main products of the reaction being cyclopentanecarboxyaldehyde (CPCA) and DHC.^46^ In general, LE can be converted in the presence of acidic heterogeneous catalysts.^23^ Amorphous silica-alumina gave CPCA and DHC with selectivities up to 77% and 7% (room temperature, 1 h and toluene as the solvent), respectively, indicating that CPCA formation is favored on the Lewis acid sites.^47^ On the other hand, carvenone was produced from LE over montmorillonite under solvent-free conditions, at a high temperature (140 °C) and short reaction time (60 min), with a yield of *ca.* 80%. Additionally, the same yield can be obtained using microwave-assisted heating in only 6 min.^48^

Heteropolyacids of the Keggin type, which possess Brønsted acid sites, were investigated in the isomerization of limonene epoxide, leading to DHC as the main product with a yield of 82%, in the presence of a toxic and rigorously regulated aprotic solvent (1,4-dioxane) under ambient conditions.^49^ The nature of the aprotic solvent (dichloromethane and 1,2-dichloroethane besides dioxane) was found to significantly affect both the reaction rate and selectivity towards DHC. A similar catalyst, silica-supported tungstophosphoric acid, demonstrated the potential to produce DHC and carvenone with yields up to 90% by adjusting the reaction conditions (although with temperatures below 40 °C in all cases) using dimethylcarbonate and diethylcarbonate as benign and green solvents.^23^ Recently, a Fe-containing ordered mesoporous catalyst (Fe/SBA-15) was tested in the reaction, showing that the product distribution (DHC, limonene-1,2-diol, and *trans*-carveol) depends on the polarity of the solvent.^50^ It was reported that an increase in polarity appears to enhance the selectivity to DHC, while limonene-1,2-diol is favored with solvents of lower polarity. Nevertheless, at the tested reaction conditions, the limonene epoxide conversion was lower than 30%, and the selectivity to isomers did not exceed 50%. Competition between isomerization and hydrolysis (Fig. 1) is expected to be one of the most important factors to control. The remaining water in the system (moisture in the solvent or the catalyst) can produce limonene-1,2-diol rather than typical isomers.^51^ Although this diol finds applications in fine chemistry, the formation of the isomers is more attractive due to their higher commercial value and direct implications in chemical processes. Selectivity depends on the specific reaction conditions as well as on the catalyst and solvent type. In general, the most critical factors influencing selectivity towards a specific target from limonene epoxide are the type of acid sites (Brønsted, Lewis, or their ratio) and the solvent (polar or non-polar).

Zeolites have garnered significant interest over the last decades in numerous fields, such as catalysis, adsorption, gas purification, wastewater treatment, and biomedical applications, among others.^52,53^ ZSM-5 is one of the most relevant zeolites due to its exceptional properties and versatility in industrial applications. However, a relatively small size of its micropores restricts the access of bulky molecules to the active sites leading to strong steric and diffusional hindrances in many applications.^54^ In this way, recent research has focused on developing ZSM-5 zeolites with improved accessibility, typically by generating a secondary porosity in the mesopore range. Interestingly, in a recent work, the synthesis of ZSM-5 zeolite showing a dendritic nanoarchitecture has been reported by Serrano *et al.*^55^ The zeolitic materials obtained in this way display 3D branched and radially oriented superstructures characterized by exceptional accessibility, attributed to the existence of a highly interconnected network of pores encompassing different scales.^55^ Therefore, dendritic ZSM-5 zeolites are expected to overcome the drawbacks inherent to conventional materials, paving the way of exploiting high porosity and accessibility of dendritic zeolites in a large variety of reactions involving bulky species.

Hence, this study aims to evaluate the use of dendritic ZSM-5 zeolite-based catalysts, in comparison with conventional and hierarchical samples, as highly selective catalysts for the isomerization of limonene-1,2-epoxide towards *cis*/*trans*-dihydrocarvone, using mild reaction conditions and more benign solvents than previously reported in the literature, such as ethyl acetate and dimethyl carbonate. The focus of this contribution was to gain an understanding of how the physicochemical properties of the zeolites are correlated with their catalytic performance in limonene-1,2-epoxide isomerization. Moreover, this study investigated the effects of substrate (*cis*-LE, *trans*-LE, and mixture-LE), solvent polarity, and catalyst robustness, and extended the scope of catalytic performance with other epoxides as substrates, such as α- and β-pinene epoxides.

## 2. Experimental section

### 2.1. Reagents

Commercial reagents were used in the experiments without further processing. Reagents for the synthesis of hierarchical and dendritic materials were aluminum isopropoxide (AlP, Sigma-Aldrich, 98 wt%); tetrapropylammonium hydroxide (TPAOH, Sigma-Aldrich, 40 wt% in water); tetraethyl orthosilicate (TEOS, Sigma-Aldrich, 98 wt%); dimethyloctadecyl [3-(trimethoxysilyl) propil] ammonium chloride (TPOAC, Sigma-Aldrich, 42 wt% in methanol); *N*-[3-(trimethoxysilyl)propyl]aniline (Ph-A, Sigma-Aldrich, 98%). Commercial ZSM-5 catalyst in H^+^ form was purchased from Zeolyst (H-CVB-80) and used as a reference (coded as ZSM-5). Reagents for the catalytic tests were (+)-limonene-1,2-epoxide (mixture of *cis*/*trans*-isomers, ≥97 wt%, Sigma-Aldrich), *cis*-(−)-limonene-1,2-epoxide (98 wt%, Sigma-Aldrich), *trans*-limonene-1,2-epoxide (97.5 wt%, Sigma-Aldrich), α-pinene epoxide (97 wt%, Sigma-Aldrich), β-pinene epoxide (80 wt%, International Laboratory USA), dimethyl carbonate (DMC, 99 wt%, Sigma-Aldrich), ethyl acetate (anhydrous, 99.8 wt%, Sigma-Aldrich), toluene (grade for liquid chromatography, Merck), acetonitrile (CH_3_CN, gradient grade for liquid chromatography, Merck), and nitrogen (99.999%, Woikoski). Other reagents used as standards for the quantification through the multipoint calibration curves were d-dihydrocarvone (≥97 wt%, mixture of isomers, Sigma-Aldrich), l-carveol (mixture of *cis* and *trans*, ≥95 wt%, Sigma-Aldrich), (1*S*,2*S*,4*R*)-(+)-limonene-1,2-diol (≥97 wt%, Sigma-Aldrich), *p*-cymene (≥90 wt%, Fluka), campholenic aldehyde (97 wt%, Hangzhou Grascent Co.), (1*R*)-(−)-myrtenal (Kosher, 97 wt%, SAFC), (*S*)-(−)-perillyl alcohol (≥90 wt%, SAFC), and (1*R*)-(−)-myrtenol (Kosher, ≥95 wt%, SAFC).

### 2.2. Synthesis of catalysts

Dendritic and hierarchical ZSM-5 materials were prepared based on the experimental methodology reported previously.^55,56^ Briefly, dendritic ZSM-5 samples were synthesized first by mixing fined milled AlP with TPAOH and distilled water at 300 rpm and room temperature, in a round bottom flask until complete dissolution of AlP. Then, a proper amount of silicon source (TEOS) was added dropwise submerging the flask in an ice bath, being then stirred at room temperature for 42 h until complete hydrolysis of TEOS. The molar composition of the initial precursor solution was 1 Al_2_O_3_ : 60 SiO_2_ : 11 TPAOH : 1500 H_2_O. Then, the alcohols produced as hydrolysis by-products were removed using a rotatory evaporator at 100 mbar and 50 °C. The clear precursor solution was pre-crystallized under reflux and stirred at 300 rpm and 90 °C for 20 h. Subsequently, the round bottom flask was cooled in an ice bath and a 5 mol% of TPOAC regarding to the initial Si content was added dropwise to the synthesis gel. The mixture was maintained under stirring in the ice bath for 6 hours. After that, the hydrothermal crystallization of the synthesis gel was performed loading it in a Teflon-lined reactor. The sealed reactor was subjected to 150 °C for two different times (4 and 7 days). After crystallization, the autoclaves were cooled down by immersion into an ice bath to suddenly interrupt the process. Two solid phases from the dendritic synthesis gel were obtained after 4 and 7 days of crystallization: a whitish supernatant phase and a white solid in the bottom of the Teflon container. The white solid phase contained the dendritic zeolite sample, and it was mechanically separated, washed with distilled water, centrifuged at 11 000 rpm three times, and dried at 100 °C overnight. The obtained solid was designated as d-ZSM-5/*X*d where d refers to the dendritic zeolite and *X* indicates the crystallization time in days (4 or 7).

A similar procedure was performed to obtain the hierarchical zeolite with some modifications. In this case, once the pre-crystallization was completed, 5 mol% of Ph-A was added to the synthesis gel, maintained at 90 °C, and stirred under reflux for 6 h. Also, to obtain the hierarchical sample, its synthesis gel was hydrothermally crystallized at 170 °C for 7 days. After which a solid phase and a transparent supernatant were obtained. The solid phase was recovered following the same procedure as for the dendritic samples and designated as h-ZSM-5.

Both hierarchical and dendritic samples were calcined in a two-step process using Ar and synthetic air as carrier gases for the first and second steps, respectively, following the procedure reported elsewhere,^57^ to prevent generation of hot spots during combustion of the organic components present in the as-synthesized zeolites.

After crystallization of the dendritic ZSM-5 samples, three different phases are obtained: the solid down phase in the synthesis reactor (*i.e.* the dendritic ZSM-5 material), the solid upper phase in the synthesis reactor (*i.e.* nano-crystalline ZSM-5 material), and the liquid solution obtained by water washing of the two former phases. At the commercial scale, the nano-crystalline ZSM-5 solid would be a co-product of the crystallization process having also practical applications. The share between both zeolitic products varies along the crystallization process, hence the dendritic ZSM-5 represents about 80% and 60% of the overall ZSM-5 yield in the synthesis performed with duration of 4 and 7 days, respectively.

CHON and TGA analyses of the solid zeolitic samples allow their content in organic components (TPA^+^ and TPOAC species) to be determined. Based on these results and considering the yield of the solid phases, it has been possible to estimate that the overall (Si + Al) atom economy during the transformation of the amorphous gel into the crystalline zeolite samples is 77%, distributed as 45% and 32% for the down and upper zeolitic phases, respectively. The remaining raw inorganic components (23%) present as soluble aluminosilicate species could be recovered in an industrial process and reused in subsequent crystallization batches. Regarding the organic reagents, a substantial part of them can also be recovered and reused after water washing and ethanol extraction treatment of the zeolites. The organic components that cannot be recovered are those strongly trapped within the zeolitic materials, being finally removed by combustion. The non-recovered (combusted) organic species represent about 16% of TPA^+^ and 36% of TPOAC of the amounts added to the synthesis gel. Accordingly, the difference (84% TPA^+^ and 64% TPOAC) could be used back in the crystallization process. Finally, the alcohols (mainly ethanol containing some isopropanol) released from the TEOS and AIP hydrolysis, are recovered by vacuum evaporation, subsequently they could also find applications when scaling up the process, thus contributing to minimizing the generation of waste streams in agreement with the green chemistry principles.

### 2.3. Catalysts characterization

X-ray diffraction patterns of calcined samples were recorded covering 2*θ* range between 0° and 5° for low-angle, and between 5° and 50° for wide angle with an Empyrean PANalytical diffractometer using Cu (Kα = 1.54 Å). Argon (−186 °C) adsorption–desorption isotherms were measured in a 3Flex instrument. Calcined samples were outgassed under vacuum at 300 °C for 5 h before adsorption analyses. The NL-DFT model was employed for calculating the pore size distribution and the cumulative pore volume over the isotherm adsorption branch. Also, the specific surface area was obtained by applying the BET model. The total pore volume (*V*_T_) was estimated at the isotherm final relative pressure (0.99), and the micropore volume (*V*_mic_) was calculated from the NL-DFT cumulative pore volume data. Finally, the external volume (*V*_ext_) was determined as the subtraction of *V*_T_ and *V*_mic_. The mesopore/external surface area (*S*_MES–EXT_) was determined as a difference between the *S*_BET_ and the microporous surface area *S*_MIC_. The latter was calculated following the procedure described elsewhere based on the application of the NL-DFT model.^58^ In addition, the BJH method was applied, using the Harkins and Jura equation, to the adsorption branch of the dendritic zeolite isotherms.

Micrographs of the calcined zeolite samples were captured using JEOL JEM 1400 transmission electron microscopes (TEM) at 120 kV. Moreover, high-resolution Transmission Electron Microscopy (HR-TEM) and high-angle annular dark field scanning TEM (HAADF-STEM) images were collected using a JEOL F200 CF (200 kV) microscope. To quantify the Si/Al ratio of the calcined zeolites, ICP-OES analyses were performed using a PerkinElmer Optima 7300 DV equipment. Calcined samples were previously subjected to sealed acid digestion with an HNO_3_ and HF solution (2 : 1 v/v) in an Anton Paar Multi-wave 3000 equipment. Solid-state ^27^Al MAS NMR spectra of the calcined zeolite samples were obtained at 104.26 MHz in a Bruker Avance III/HD 400 MHz spectrometer.

To determine the concentrations of Brønsted and Lewis acid sites (BAS and LAS, respectively), pyridine served as a probe molecule, and the assessment was conducted through FTIR in a custom-built system. Self-supported wafers, weighing 15 mg cm^−2^, were fabricated and subjected to activation under a vacuum (10^−4^ mbar) at 525 °C for 1 h before measurements. Subsequently, pyridine was introduced into the system at 150 °C, and it was sealed for 20 minutes. Thermal desorption was performed under high vacuum, with temperatures escalating within the range of 150 to 450 °C (heating rate: 10 °C min^−1^), including a 20 min equilibrium period before obtaining the spectrum at each designated temperature. Spectra were recorded utilizing a Jasco-4600 instrument equipped with a TGS detector, with a resolution of 4 cm^−1^ and 128 scans. The molar extinction coefficients used for quantifying BAS and LAS concentrations were sourced from Zholobenko *et al.*^59^ for the ZSM-5 zeolite: *ξ*_BAS_ = 1.09 cm μmol^−1^ and *ξ*_LAS_ = 1.71 cm μmol^−1^. Similarly, the concentration of external Brønsted acid sites was assessed by adsorbing 2,6-di-*tert*-butylpyridine (DTBPyr) as a probe molecule (*ε*_Bext_ = 5.3 cm μmol^−1^),^60^ following the same procedure described earlier and maintaining adsorption–desorption equilibrium intervals of 60 minutes. The spent catalysts were characterized using thermogravimetric analysis (TGA) with a NETZSCH STA 449 thermobalance. These assays were performed under air flow and heating the samples to 900 °C at 10 °C min^−1^, followed by an isothermal step of 10 min.

### 2.4. Catalytic tests

The isomerization of limonene-1,2-epoxide over ZSM-5 zeolites was performed in a batch glass reactor with a total liquid volume of 75 mL and under a nitrogen atmosphere. The flask was equipped with a thermocouple, a N_2_ feeding tube, a sampling valve, and a condenser (Fig. S1†). Catalytic tests were performed using a sufficiently high liquid volume-to-catalyst mass ratio and vigorous agitation (520–530 rpm) to overcome external mass-transfer limitations.^61,62^ Furthermore, small catalyst particles (<75 μm) were used to suppress the internal mass transfer limitations. In a typical experiment, 115 mg of catalyst was heated at 250 °C for 30 min under a N_2_ atmosphere to ensure the removal of physisorbed water. Subsequently, 152 mg (1 mmol) of epoxide was added to the pre-heated solvent at 70 °C to reach 75 mL as the total liquid volume. Samples of approximately 0.4 mL were collected at various time intervals using a syringe equipped with 0.45 μm filters to analyze the reaction kinetics using an Agilent Technologies GC-6890N equipment provided with a DB-1 column (30 m length × 250 μm internal diameter × 0.50 μm film thickness), a FID detector, and an autosampler. Helium was used as the carrier gas (1.5 mL min^−1^, 0.12 MPa) with a split ratio of 10 : 1. The detector temperature was set to 280 °C, and the injection volume was 1 μL. The oven-temperature program ramped from 60 °C to 100 °C at a rate of 20 °C min^−1^, followed by an increase to 200 °C at 10 °C min^−1^, this temperature being held for 1 min. The nature of the products was further confirmed with an Agilent GC/MS 6890N/5973N equipped with a DB-1 capillary column (30 m length × 250 μm internal diameter × 0.5 μm film thickness).

The limonene-1,2-epoxide conversion (*X*_LE_), the selectivity to the product i (*S*_i_), and the yield to the product (*Y*_i_) were calculated based on the eqn (1)–(3).1
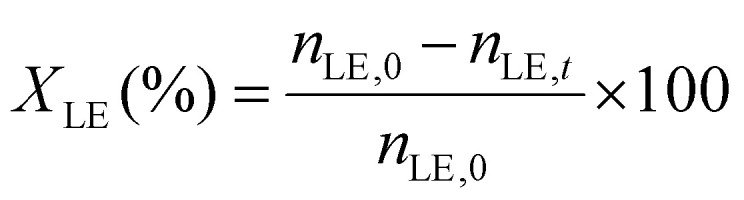
2
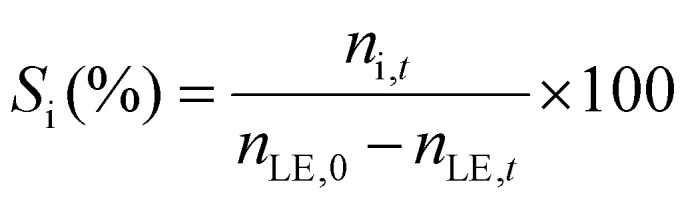
3
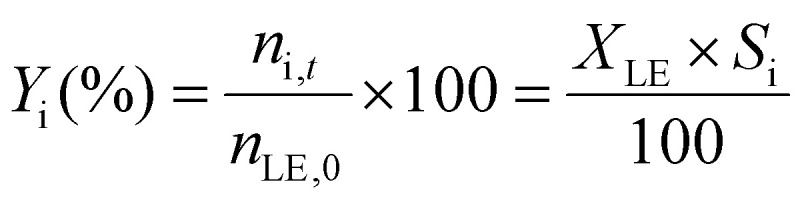
where *n*_LE,0_, *n*_LE,*t*_, and *n*_i,*t*_ refer to the initial moles of epoxide, the moles of epoxide after time *t*, and the moles of the product i after time *t* in the reaction mixture, respectively. The concentrations of limonene-1,2-epoxide as the substrate, isomeric products such as dihydrocarvone and carveol, and limonene-1,2-diol as the hydration product were determined from multipoint calibration curves.

The initial reaction rate and TOF for limonene-1,2-epoxide conversion were calculated using eqn (4) and (5):4
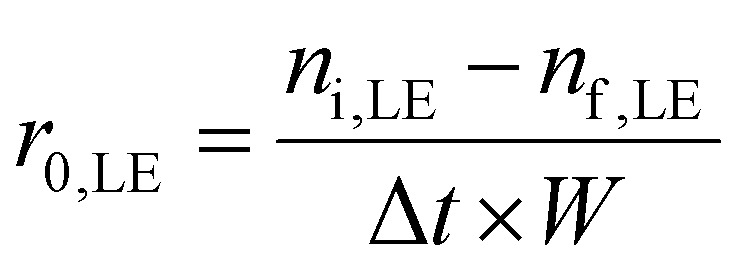
5
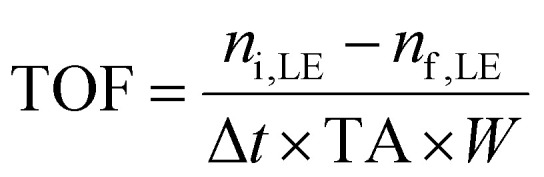
where *n*_i,LE_ and *n*_f,LE_ correspond to moles of limonene-1,2-epoxide at time 0 min and 5 min, respectively, Δ*t* is the time interval, *W* is the catalyst weight, and TA is the total acidity referred to the sum of Brønsted and Lewis acidity of the catalyst.

## 3. Results and discussion

### 3.1. Catalyst characterization

#### 3.1.1. X-ray diffraction

**Fig. 2:**
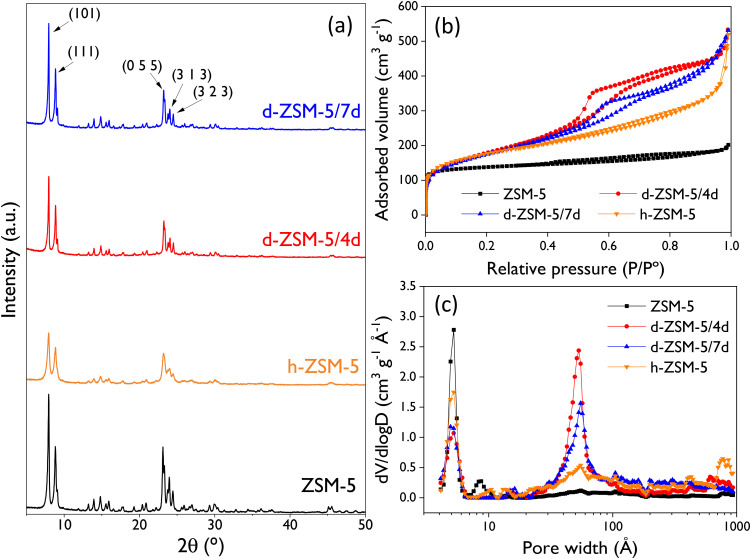
(a) XRD patterns of the ZSM-5 samples, and textural properties by Ar physisorption at 87 K of the ZSM-5 samples: (b) adsorption–desorption isotherms and (c) NL-DFT pore size distribution.

X-ray diffraction patterns of calcined ZSM-5 catalyst are shown in Fig. 2A. All samples display the characteristic peaks of a well-crystallized MFI zeolitic structure.^63^ However, conventional ZSM-5 zeolite shows the highest diffraction intensity due to the larger zeolitic domains of the commercial sample compared with the synthesized materials, which are formed by aggregates of very small zeolite nanounits.^56,64^ A slight enhancement in the diffraction intensity is also observed for the dendritic samples when the crystallization time is increased from 4 to 7 days. In addition, to probe the presence of porosity in the mesoscale, low-angle X-ray diffraction was performed over hierarchical and dendritic samples (Fig. S2†). A broad diffraction signal is detected for the d-ZSM-5/4d sample between 1.2° and 2.2°, suggesting the presence of some degree of mesoscopic order in the sample obtained after 4 days of hydrothermal crystallization, that disappears when increasing the crystallization time up to 7 days. This signal is not detected for the h-ZSM-5 sample, being in good agreement with previous work.^64^

#### 3.1.2. Argon physisorption

Fig. 2B and C display the argon adsorption–desorption isotherms and the pore size distribution, respectively. The reference ZSM-5 zeolite exhibits a type I(a) isotherm, characteristic of microporous materials.^65^ On the other hand, h-ZSM-5 and d-ZSM-5 samples present isotherms with contributions from both micro- and mesopores, but with significant differences among them. The hierarchical ZSM-5 zeolite (h-ZSM-5) shows adsorption in the range *P*/*P*_0_ = 0.2–0.9 due to the presence of a broad mesoporosity with pore sizes between 20 and 200 Å, as illustrated in Fig. 2C. These findings align well with other hierarchical samples synthesized under similar conditions.^56^ Concerning the dendritic zeolites, both samples exhibit hybrid type I(a) and IV(a) isotherms due to the presence of well-defined micro- and mesopores. Notably, there are differences in the mesopore size distribution between them. The d-ZSM-5/4d sample displays a narrower distribution centered at 51.4 Å, whereas the d-ZSM-5/7d sample exhibits less uniform mesopores centered at 61 Å (both sizes being determined by applying the BJH model to the adsorption branch of the isotherms). This fact indicates variations in the mesoporosity during the crystallization process at 150 °C, as previously reported.^55^

The textural properties of the catalysts (Table 1) are in good agreement with the pore size distributions and cumulative pore volumes calculated with the NL-DFT model. In general, both the BET and the mesopore/external surface areas increase with the mesoporosity share. As expected, a direct correlation exists between the reduction in micropore volume (*V*_mic_) and the extent of mesoporosity introduced in the zeolitic structure. In this context, the conventional ZSM-5 sample exhibits the highest micropore volume (0.201 cm^3^ g^−1^), whereas the most extensively modified catalyst, the d-ZSM-5/4d zeolite, displays the lowest microporosity (0.131 cm^3^ g^−1^) and the highest BET and mesopore/external surface areas (570 and 360 m^2^ g^−1^, respectively). It is also remarkable that the dendritic samples exhibit the highest levels of mesoporosity share (*ca.* 80%). This underscores the exceptional accessibility of these samples, being much higher than the pore volume of the conventional ZSM-5 sample. Therefore, it is expected that these large differences in terms of textural properties and accessibility between the four ZSM-5 samples will have a strong effect on their catalytic behavior in reactions that may suffer from strong steric and/or transport limitations due to the bulky size of the involved species, as it is the case of the limonene-1,2-epoxide isomerization herein investigated.

**Table 1:** Al content and textural properties of the ZSM-5 samples

Catalyst	Si/Al	*S*_BET_ (m^2^ g^−1^)	*S*_MES/EXT_ (m^2^ g^−1^)	*V*_mic_ (cm^3^ g^−1^)	*V*_T_ (cm^3^ g^−1^)	Mesoporosity^a^ (%)
ZSM-5	40	426	77	0.20	0.26	21.8
h-ZSM-5	40	557	279	0.17	0.66	73.8
d-ZSM-5/4d	42	570	360	0.13	0.68	80.7
d-ZSM-5/7d	41	553	330	0.14	0.68	79.5

a Calculated as *V*_Ext_/*V*_T_ × 100.

#### 3.1.3. Catalyst acidity

Table 2 presents the acid properties of the calcined catalysts as determined by pyridine-FTIR measurements. Although all the samples present quite similar Al content (Table 1), significant differences can be appreciated in their acidic features. Specifically, both ZSM-5 and h-ZSM-5 samples exhibit a high concentration of Brønsted acid sites. Conversely, the dendritic ZSM-5 materials show a substantial reduction in the Brønsted acid site population at the expense of an increase in that of Lewis acid sites. These findings establish a trend in the BA/LA concentration ratio as follows: ZSM-5 > h-ZSM-5 > d-ZSM-5/7d > d-ZSM-5/4d, mirroring the mesoporosity and mesopore/external surface area contributions outlined in Table 1. Interestingly, the large concentration of Lewis acid sites in the dendritic samples can be mainly related with Al framework species since ^27^Al MAS NMR measurements indicate that the share of extra-framework Al atoms is just about 6–7% for both d-ZSM-5 samples. Another remarkable finding is the high concentration of external Brønsted acid sites for the dendritic materials, and, in particular, for the d-ZSM-5/4d sample, denoting the presence of a highly accessible acidity.

**Table 2:** Acidic properties of the ZSM-5 samples determined with pyridine-FTIR

Catalyst	Brønsted acidity (μmol g^−1^)				Lewis acidity (μmol g^−1^)				Total (μmol g^−1^)	BA/LA ratio	*C*_B,ext_ (μmol g^−1^)
	Weak	Medium	Strong	Total	Weak	Medium	Strong	Total			
ZSM-5	40	52	97	189	10	7	14	31	220	6.1	16
h-ZSM-5	47	53	99	199	11	5	49	65	264	3.1	41
d-ZSM-5/4d	33	32	58	123	21	9	58	88	211	1.4	97
d-ZSM-5/7d	32	34	65	131	22	11	53	86	217	1.5	61

#### 3.1.4. TEM analyses

**Fig. 3:**
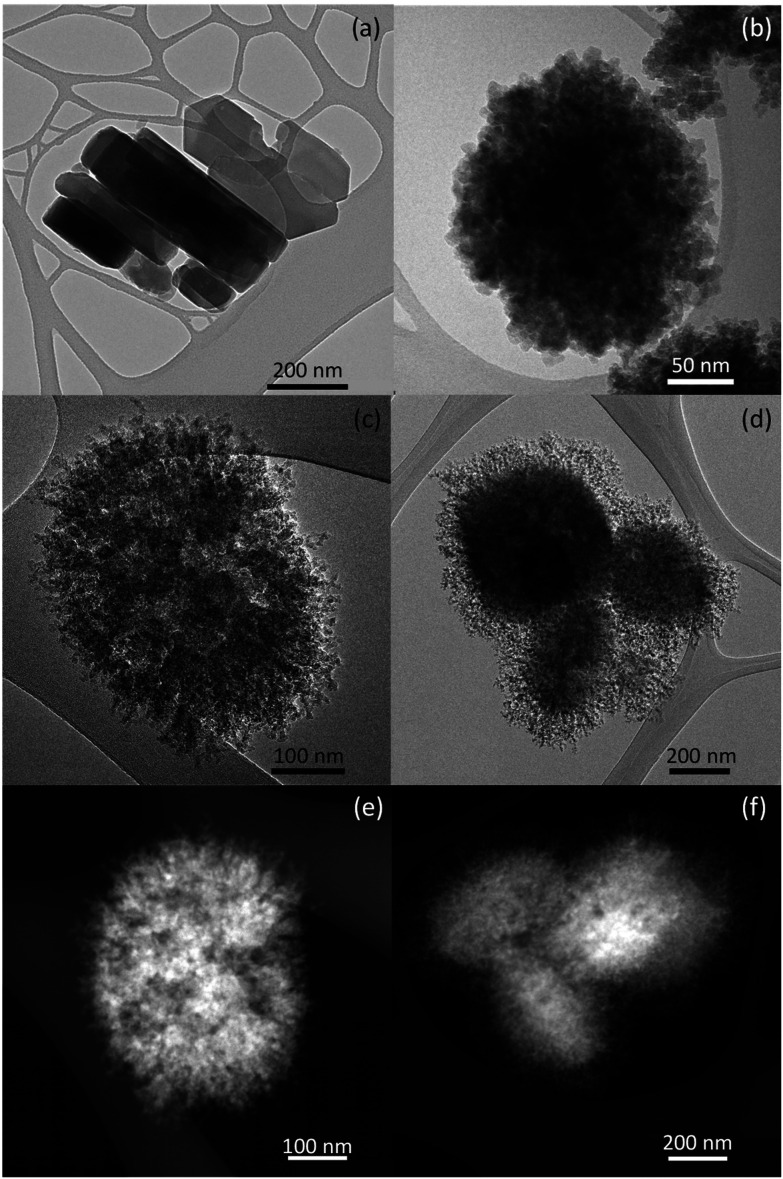
TEM images of ZSM-5 (a), and h-ZSM-5 (b) samples. HR-TEM and HAADF-STEM micrographs of d-ZSM-5/4d (c, e), and d-ZSM-5/7d (d, f) samples.

TEM micrographs in Fig. 3 highlight distinct morphological variations among the calcined catalysts. The conventional ZSM-5 sample exhibits the characteristic coffin-like particles of the MFI structure, ranging in size from 70–100 nm for the smallest dimension to approximately 400 nm for the largest one. In contrast, the h-ZSM-5 sample consists of 250–300 nm-sized globular aggregates composed of nanoparticles measuring about 5–10 nm. This random aggregative structure creates mesopores with a broad pore size distribution. On the other hand, the dendritic samples showcase oval-shaped particles measuring *ca.* 400 nm × 250 nm. These particles are formed by nanounits of about 5 nm, arranged in rod-like spikes that extend from the inner to the outer part of the particle, giving rise to the radially oriented and branched morphology distinctive of dendritic ZSM-5 zeolites. Notably, some large vesicles, previously reported for this morphology,^55^ are also visible within the particles of both d-ZSM-5 samples in the HAADF-STEM images. All these features denote the high connectivity existing between the different types of porosities present in the d-ZSM-5 samples.

### 3.2. Catalytic activity

#### 3.2.1. General aspects

Fig. S3† shows a general scheme of the products that were identified in the limonene-1,2-epoxide (LE) isomerization over the ZSM-5 catalysts, namely *cis*-dihydrocarvone (1a), *trans*-dihydrocarvone (1b), carveol (2), 1,3,4-trimethyl-3-cyclohexen-1-carboxaldehyde (3, CAS number: 40702-26-9), fenchone (4), product 5, 3-methyl-2-methylidene-bicyclo[3.2.1]oct-3-ene (6, CAS number: 49826-53-1), (2*S*,4*R*)-*p*-mentha-1(7),8-dien-2-ol (7, CAS number: 2102-62-7), and limonene diol (8). Detailed information regarding retention times and mass spectra can be found in the ESI (Table S3 and Fig. S10–S15†).

Repeatability of the experiments was investigated using d-ZSM-5/4d catalyst with the results demonstrating the reliability of the procedure (Fig. S4†). Additionally, Fig. S5† unequivocally confirms that the transformations follow a catalytic route, as evidenced by the linear dependence observed between the initial reaction rate and the catalyst mass.

#### 3.2.2. Turnover frequency (TOF)

**Fig. 4:**
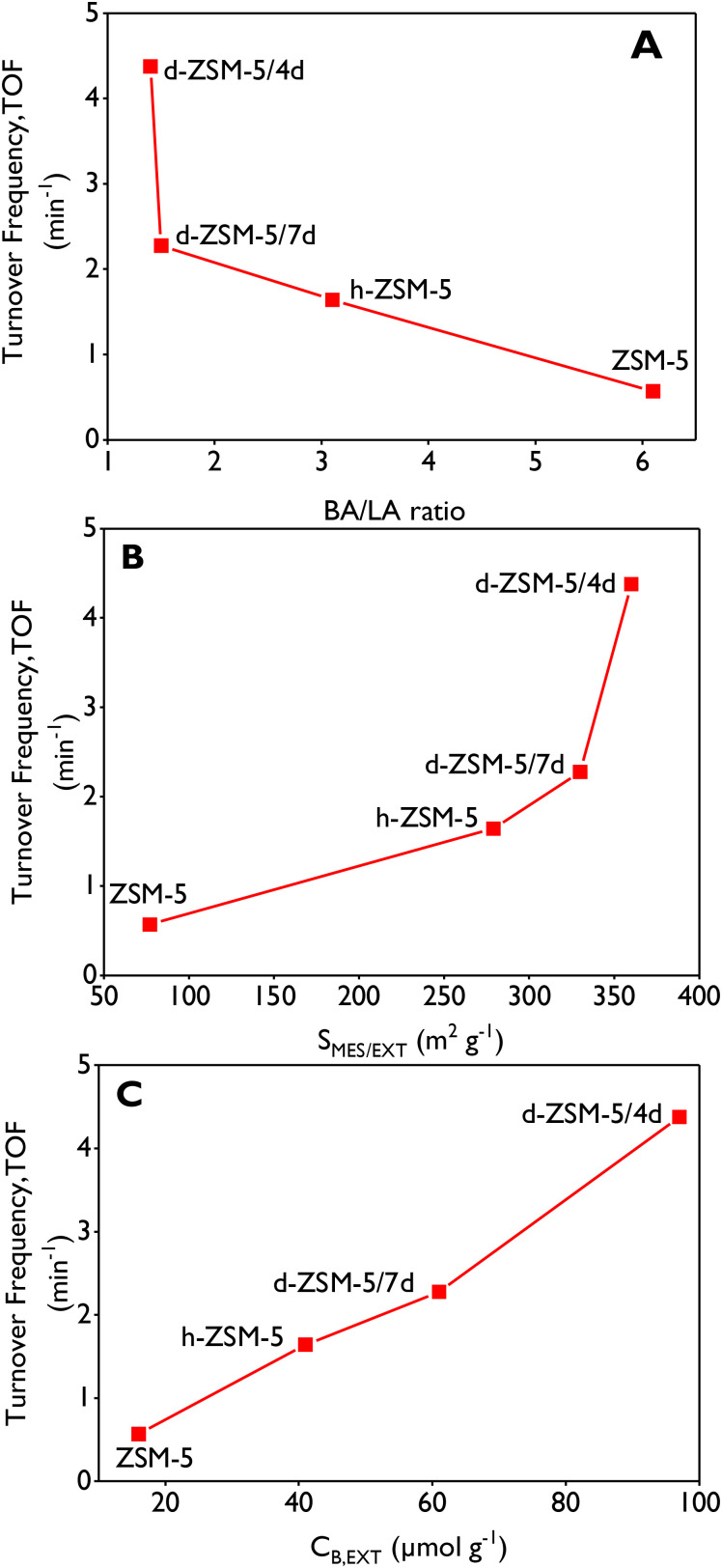
Turnover frequency (TOF) of limonene-1,2-epoxide as a function of (A) BA/LA ratio, (B) external/mesopore surface area and (C) external Brønsted acidity. Reaction conditions: *C*_LE,0_ = 13 mmol L^−1^, 75 mL of total volume, anhydrous ethyl acetate as a solvent, 115 mg of catalyst, 70 °C, 520–530 rpm, N_2_ atmosphere.

TOF values of limonene-1,2-epoxide (LE) have been correlated against the Brønsted/Lewis (BA/LA) molar ratio (Fig. 4A), the external/mesopore surface area (Fig. 4B) and the external Brønsted acid site concentration (Fig. 4C). The highest LE isomerization TOF was obtained with d-ZSM-5/4d catalyst, which exhibits the lowest BA/LA ratio (1.4), the largest mesoporosity contribution but also the highest share of external Brønsted acid sites. Thus, d-ZSM-5/4d sample exhibits a TOF value sevenfold higher than that of the conventional ZSM-5 material. The plots suggest a dependency between TOF and the external/mesopore surface area but the opposite effect with the BA/LA ratio. Moreover, a very good correlation is observed in Fig. 4C between the catalytic activity and the external Brønsted acid site density. This relevant finding confirms high significance of steric and diffusional limitations due to the large molecular size of the different species involved in the reaction regarding the ZSM-5 micropores. Therefore, it can be concluded that remarkable accessibility of the dendritic ZSM-5 materials contributes to overcoming those hindrances. In this way, Table S1† illustrates a comparison based on TOF values for the four zeolites tested in this work with some recently reported heterogeneous systems, demonstrating superior activity in the isomerization of limonene-1,2-epoxide obtained with both dendritic ZSM-5 zeolites (4.4 and 2.3 min^−1^ for d-ZSM-5/4d and d-ZSM-5/7d, respectively).

#### 3.2.3. Conversion after prolonged times and product distribution

**Fig. 5:**
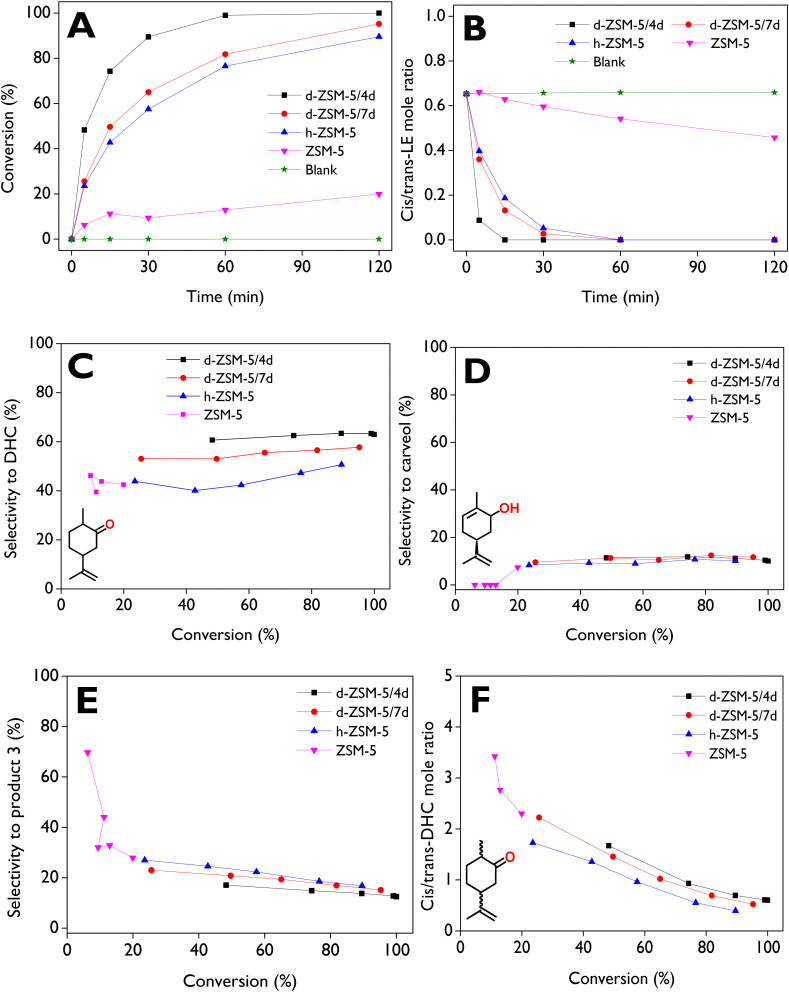
(A) Limonene-1,2-epoxide conversion and (B) *cis*/*trans* limonene-1,2-epoxide molar ratio as a function of the reaction time, (C) selectivity to dihydrocarvone, (D) selectivity to carveol, (E) selectivity to product 3, and (F) *cis*/*trans* dihydrocarvone molar ratio, as a function of the conversion. Reaction conditions: *C*_LE,0_ = 13 mmol L^−1^, 75 mL of total volume, anhydrous ethyl acetate as a solvent, 115 mg of catalyst, 70 °C, 520–530 rpm, N_2_ atmosphere.

The conversion and selectivity towards target products in the isomerization of LE significantly depend on the zeolite nanoarchitecture, as well as on their acidic and textural properties. Fig. 5A illustrates the conversion profiles for the commercial microporous zeolite (ZSM-5), the hierarchical zeolite (h-ZSM-5), and the two dendritic-based ZSM-5 catalysts. Furthermore, the reaction was conducted without a catalyst to demonstrate that it did not proceed solely due to the thermal effect (Blank, Fig. 5A), This is consistent with our earlier conclusions based on the linear dependence observed between the initial reaction rate and the catalyst mass. ZSM-5 exhibited limited reactivity for the transformation of LE, achieving only 20% conversion after 2 h, which could be assigned mainly to its small pore size (5.5 Å) since it is significantly lower than the LE kinetic diameter (8.35 Å, estimated by DFT). Accordingly, it can be assumed that LE is strongly hindered from entering the MFI micropores, hence the acid sites located on the external/mesopore surface area or at the pore mouths would be mainly responsible for the observed catalytic activity, which agrees well with the direct relationship between TOF and external Brønsted acidity shown earlier. Thus, when the external/mesopore surface area (Table 1) is increased by the creation of mesopores, the conversion is significantly sharply enhanced, reaching around 90, 95 and 100% with h-ZSM-5, d-ZSM-5/7d and d-ZSM-5/4d catalysts, respectively, after 2 h.

The evolution of the *cis*/*trans*-LE molar ratio as a function of time is displayed in Fig. 5B for all catalysts, with an initial value around 0.65, corresponding to a molar composition of 38% *cis*-LE and 59% *trans*-LE in the commercial reagent. With the dendritic zeolites, values close to zero are obtained after 30 min while, with ZSM-5, a value of *ca.* 0.45 is reached after 2 h due to its low catalytic activity. Furthermore, the appreciable drop observed with dendritic zeolites in only 15 min allows to conclude that the reactivity of *cis*-LE is higher. The same conclusions can be drawn from the plots of the conversion of each isomer (*cis*-LE and *trans*-LE) as a function of reaction time, which are displayed in Fig. S7A and B in the ESI.† A larger steric hindrance of the oxygen atom in the *trans*-LE, in comparison with *cis*-LE, can explain the lower reactivity of *trans*-LE, as it is more difficult for this molecule to access the active sites of the catalyst.^23^

The highest selectivity to DHC (*cis* + *trans*) was achieved with d-ZSM-5/4d (Fig. 5C), showing a small variation range (60–63%) during the reaction. Lower values were obtained with d-ZSM-5/7d (53–57%), h-ZSM-5 (40–50%), and conventional ZSM-5 samples. This is also an interesting fact as it indicates that the zeolite catalyst showing the highest activity is also the most selective for DHC formation. Selectivity to carveol (*cis* + *trans*) for all catalysts is displayed in Fig. 5D, with constant values around 10–12% over a wide conversion range for the hierarchical and the two dendritic zeolites. This suggests a minor effect of the BA/LA ratio and textural properties on carveol selectivity, in contrast to the selectivity for DHC. The low selectivities to carveol can be attributed to the absence of a bifunctional acid/base catalyst in the system, as reported for a catalytic mechanism converting an epoxide into an allylic alcohol by Raptis *et al.*^66^

No carveol was observed for ZSM-5 before 60 min of the reaction (up to 13% conversion). However, at the maximum conversion achieved under the tested conditions (20%), approximately 7.6% selectivity was reached. This behavior can be explained by the rapid decrease in the selectivity to product 3 (Fig. 5E) in the 0–20% conversion range, reaching about 28% at 20% conversion. This value is slightly higher than the selectivity range using the dendritic zeolites, which showed values of *ca.* 12–17% and 15–23% for d-ZSM-5/4d and d-ZSM-5/7d, respectively. The *cis*/*trans*-DHC molar ratio, depicted as a function of conversion in Fig. 5F, exhibited similar behavior across all catalysts. However, a slight preference for *cis*-DHC is observed with d-ZSM-5/4d compared to d-ZSM-5/7d. Moreover, with the dendritic zeolites, a final ratio (complete conversion) of *ca.* 0.53–0.60 is achieved.

A detailed kinetic and mechanistic study on LE isomerization over dendritic ZSM-5 exploring also the temperature dependence will be a subject of a separate investigation.^67^

The d-ZSM-5/4d catalyst exhibited a DHC yield of 63% after 2 h, significantly outperforming other catalytic systems based on amorphous silica-alumina (yield < 7% with toluene at room temperature after 1 h),^47^ montmorillonite (yield < 20% under solvent-free conditions at 140 °C after 1 h),^48^ and Fe/SBA-15 (yields < 10% using various solvents at 70 °C after 1 h).^50^ Moreover, the primary products with the first two literature catalysts were cyclopentanecarboxyaldehyde and carvenone, achieving yields of *ca.* 77% and 80%, respectively.^47,48^ For Fe/SBA-15, conversions of limonene epoxide with any solvent did not exceed 21% after 1 h.^50^ Although a combined selectivity to DHC and carvenone of *ca.* 85–90% over a heteropolyacid/SiO_2_ has been reported,^23^ which describes two consecutive steps, including the fast production of DHC from LE and the slow isomerization of DHC into carvenone, the formation of carvenone was not observed in this work.

#### 3.2.4. Effect of the substrate

**Fig. 6:**
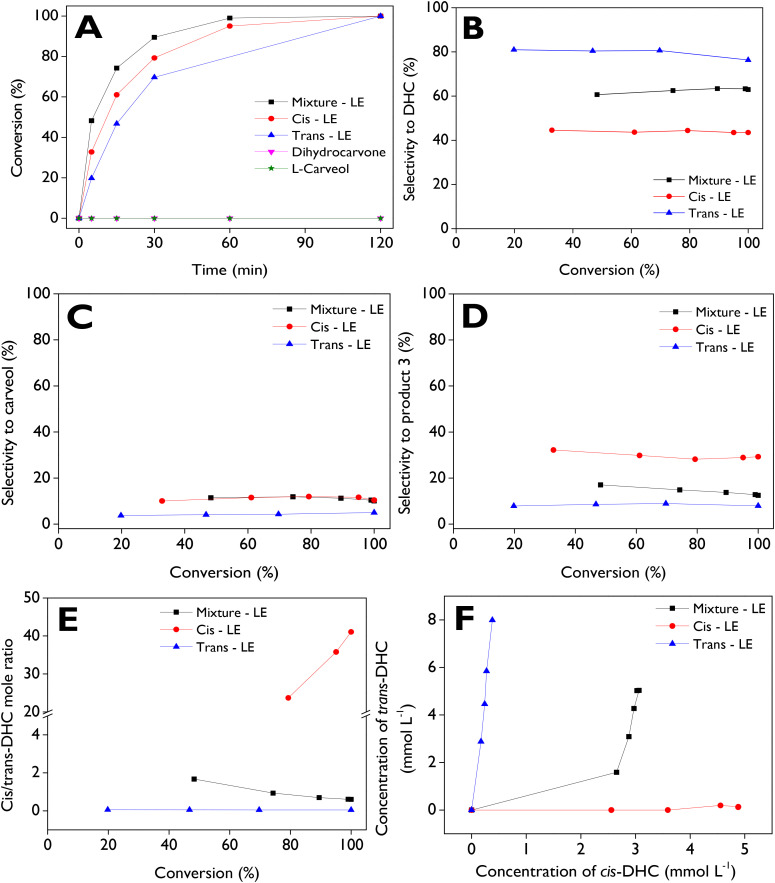
Effect of the substrate on its isomerization over d-ZSM-5/4d. (A) Substrate conversion as a function of the reaction time; (B) selectivity to dihydrocarvone, (C) selectivity to carveol, (D) selectivity to product 3, and (E) *cis*/*trans* dihydrocarvone molar ratio, as a function of the conversion; (F) concentration of *trans*-DHC *vs.* concentration of *cis*-DHC. Reaction conditions: *C*_substrate,0_ = 13 mmol L^−1^, 75 mL of total volume, anhydrous ethyl acetate as a solvent, 115 mg of d-ZSM-5/4d, 70 °C, 520–530 rpm, N_2_ atmosphere.

Due to the commercial substrate used for assessing the limonene-1,2-epoxide isomerization is a mixture of *cis* and *trans* isomers (*cis* : *trans* molar ratio of 0.65 : 1), it is advisable to evaluate how the transformation proceeds starting with pure *cis* and *trans* isomers, utilizing the most active catalyst (d-ZSM-5/4d) at 70 °C, with anhydrous ethyl acetate as the solvent. Additionally, two main products, dihydrocarvone and carveol, were employed as starting substrates to investigate their stability or, conversely, to assess if they can be transformed under the tested reaction conditions. Fig. 6A illustrates the dependence of conversion as a function of time for five substrates, with complete conversions achieved after 2 h for the three LE sources. In contrast, no activity was observed with dihydrocarvone and l-carveol as starting reagents. This leads to the conclusion that the two main products during LE isomerization over dendritic zeolites are very stable. On the other hand, the reactivity order of the substrates is as follows: mixture-LE > *cis*-LE > *trans*-LE, with initial reaction rates of 0.92, 0.54, and 0.28 mmol min^−1^ g^−1^, respectively. These results suggest a synergistic effect in the way that the mixture of isomers interacts in the catalyst surface, potentially enhancing the reaction rate and overall conversion, in comparison with the pure isomers as initial substrates.^68,69^ The comparison of the initial reaction rates of neat isomers (*cis*-LE and *trans*-LE) with the analogous values of isomers present in the mixture-LE shows values of 0.62 and 0.30 mmol min^−1^ g^−1^ for *cis*-LE and *trans*-LE, respectively. These values are slightly higher than those of the neat isomers (0.54 and 0.28 mmol min^−1^ g^−1^), confirming the synergistic effect of using the mixture-LE as raw material in the isomerization reaction.

*trans*-LE exhibits high selectivity to DHC (Fig. 6B), maintaining values of *ca.* 80% across a wide conversion range. Surprisingly, our results suggest a high favorability of the formation of *trans*-DHC from *trans*-LE, as indicated in Fig. 6E and F. In contrast, when starting with *cis*-LE, only a maximum selectivity of *ca.* 45% to DHC is achieved, with *cis*-DHC being the primary configuration produced. Fig. 6C illustrates selectivities towards carveol, indicating no significant changes in this parameter when starting with mixture-LE and *cis*-LE, reaching maximum values of *ca.* 12%, while maximum values of approximately 4% are observed with *trans*-LE. Conversely, significant formation of product 3 (Fig. 6D) is achieved with *cis*-LE as the substrate, reaching a maximum value of about 33%, whereas only maximum values of approximately 9% are reached when starting with *trans*-LE. Mixture-LE yields intermediate selectivity values ranging between 12% and 17%. *trans*-DHC and *cis*-DHC are formed in a parallel pathway, as shown in Fig. 6F.

#### 3.2.5. Solvent effects

**Fig. 7:**
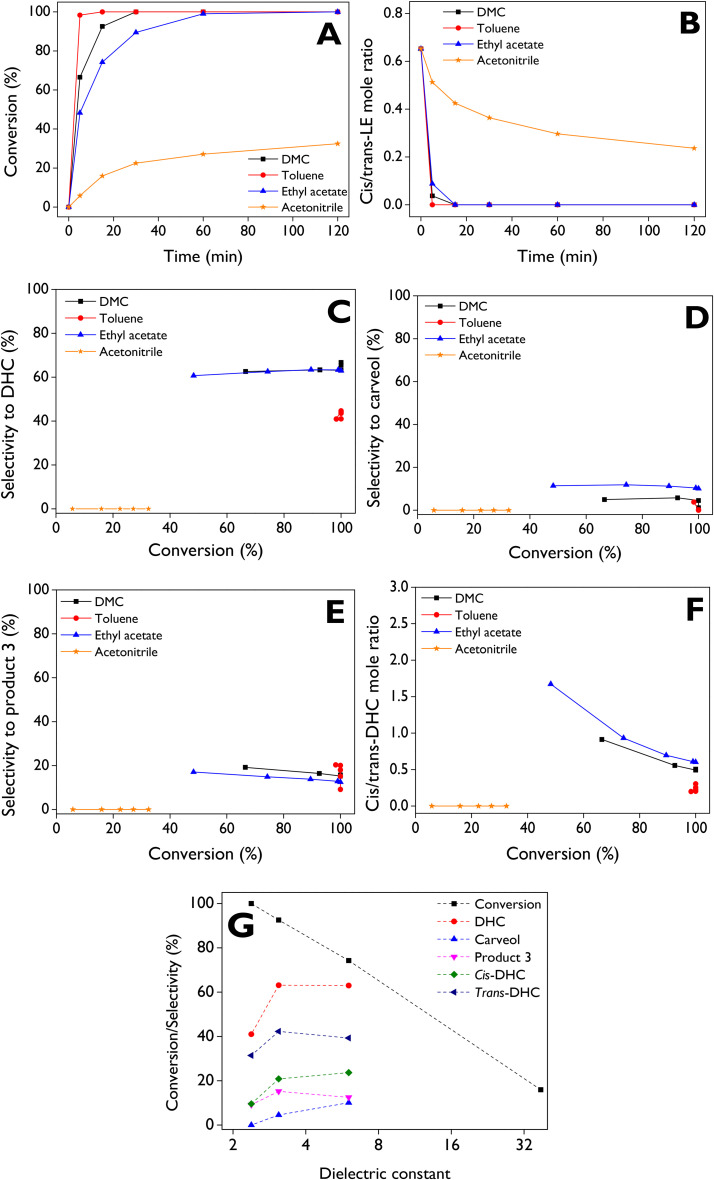
Effect of the solvent on the LE isomerization over d-ZSM-5/4d. (A) LE conversion and (B) *cis*/*trans*-LE molar ratio as a function of the reaction time; (C) selectivity to dihydrocarvone, (D) selectivity to carveol, (E) selectivity to product 3, and (F) *cis*/*trans* dihydrocarvone molar ratio, as a function of the conversion; (G) conversion after 15 min or selectivity at 100% conversion as a function of the solvent dielectric constant: toluene (*ε* = 2.38), dimethyl carbonate (*ε* = 3.09), ethyl acetate (*ε* = 6.02), acetonitrile (*ε* = 37.5). Reaction conditions: *C*_LE,0_ = 13 mmol L^−1^, 75 mL of total volume, 115 mg of d-ZSM-5/4d, 70 °C, 520–530 rpm, N_2_ atmosphere.

The solvent plays a critical role in the isomerization of limonene-1,2-epoxide, as reported in the literature^23,49,50^ because it can control the stabilization of the charge of the intermediate carbocation during the reaction.^23^ Solvents with dielectric constants between 2.2 and 3.1, such as 1,4-dioxane, toluene, and DMC, were earlier evaluated using Keggin heteropolyacids-based materials^49^ and Fe/SBA-15 as catalysts.^50^ Therefore, the effect of the solvent is here assessed using a wide range of solvent polarity, represented by its values of dielectric constant. Fig. 7A shows the conversion profiles obtained with different solvents using the d-ZSM-5/4d sample, allowing to establish the reactivity order, which increases as follows: acetonitrile < ethyl acetate < DMC < toluene, exhibiting initial reaction rates of 0.10, 0.92, 1.2, and 1.7 mmol min^−1^ g^−1^, respectively. Complete LE conversions were achieved after 15, 30, and 60 min with toluene, DMC, and ethyl acetate, respectively, whereas acetonitrile resulted in a low conversion of *ca.* 32% after 2 h. Fig. 7B shows that *cis*-LE is more reactive than *trans*-LE with solvents of low (toluene) and medium (DMC, ethyl acetate) polarity, given the *cis*/*trans*-LE ratio (Fig. 7B) achieved zero values at lower times compared to the complete conversion (Fig. 7A). In contrast, the most polar solvents, such as acetonitrile, exhibited a more competitive consumption of both *cis*-LE and *trans*-LE. The conversion profiles of the isomers, *cis*-LE and *trans*-LE, along the reaction time exhibit the expected behavior according to Fig. 7B and Fig. S7C and D in the ESI.†

A constant trend of DHC selectivity (Fig. 7C) is observed as the reaction progresses when using ethyl acetate and DMC as solvents, with values ranging from about 62% to 65%. In contrast, results with toluene indicate that LE isomerization is not favored towards DHC at high conversions, showing selectivity values between 40% and 44%. The highest (10–12%) and lowest (1–4%) selectivities to carveol (sum of *cis* and *trans*) are achieved with ethyl acetate and toluene as solvents, respectively (Fig. 7D). With DMC, selectivities to carveol are in the range of *ca.* 1 to 5%. The selectivity to product 3 (Fig. 7E) ranges between 10–20%, 15–20%, and 12–17% with toluene, DMC, and ethyl acetate, respectively.

*trans*-DHC is favored over *cis*-DHC as the reaction proceeds with different solvents (Fig. 7F), stabilizing in a *cis*/*trans* molar ratio of about 0.5–0.6 at conversions above 80% with ethyl acetate and DMC, while toluene notably favors the formation of *trans*-DHC over *cis*-DHC, reaching ratios between 0.2–0.3. Fig. 7G shows that the LE conversion can be directly linked to low solvent polarity, while selectivity to DHC (*cis* + *trans*) reaches maximum values with moderately polar solvents such as ethyl acetate and DMC. However, *cis*-DHC is favored with ethyl acetate, whereas the formation of *trans*-DHC is promoted with DMC. On the other hand, carveol is most favored in solvents with medium polarity as ethyl acetate. A highly polar solvent, such as acetonitrile, is not suitable for LE isomerization leading to low conversions, and additionally, no formation of any of the 8 products in Fig. S3† was observed (Table 3). Contrarily, a main product was identified by GC-MS with low quality (35%) having the CAS number: 20662-85-5, its mass spectra being provided in Fig. S15.†

**Table 3:** Product distribution on the isomerization of limonene-1,2-epoxide over d-ZSM-5/4d with different solvents. Notation for the different components is given in Fig. S3†

Solvent	−*r*_LE,0_ (mmol min^−1^ g^−1^)	TOF (min^−1^)	Conversion (%)	Selectivity (%)									
				1a	1b	2	3	4	5	6	7	8	Others
Toluene	1.7	7.9	100^a^	9.6	31.4	0.0	9.1	0.1	0.0	8.5	1.7	0.0	39.6
DMC	1.2	5.5	70	29.9	32.7	5.0	19.2	1.7	3.7	2.1	3.5	2.3	0.0
			100^a^	22.4	44.3	0.4	15.6	3.0	2.3	6.2	3.4	2.2	0.0
Ethyl acetate	0.9	4.4	70	30.2	32.4	11.9	14.9	1.6	3.0	1.9	4.2	0.0	0.0
			100^a^	23.7	39.3	10.1	12.5	3.3	2.3	4.9	4.0	0.0	0.0
Acetonitrile	0.1	0.5	32^a^	0.0	0.0	0.0	0.0	0.0	0.0	0.0	0.0	0.0	100.0

a Conversion values after 120 min. Reaction conditions: *C*_LE,0_ = 13 mmol L^−1^, 75 mL of total volume, 115 mg of d-ZSM-5/4d, 70 °C, 520–530 rpm, N_2_ atmosphere.

In summary, it can be concluded that the LE isomerization over the d-ZSM-5/4d catalyst is favored in non-polar solvents such as toluene, but its transformation is highly selective towards DHC in solvents with medium polarity, such as DMC and ethyl acetate. These results can be mechanistically explained by the dipolar orientations of solvents with the intermediate carbocation, facilitating the rearrangement of limonene-1,2-epoxide. Stability of this carbocation is influenced by solvent polarity, which plays a crucial role in determining product selectivity and favoring one specific route over others. Similar effects of solvent polarity have been reported for the rearrangement of other monoterpene epoxides, such as α-pinene epoxide, where it favors formation of campholenic aldehyde and carveol as the major products.^50^ The carbocation obtained after breaking the C–O bond, can produce several thermodynamic products, as demonstrated in the literature.^70^ These insights support the carbocations in the reaction mechanism of the rearrangement of limonene epoxide towards dihydrocarvone over a heteropolyacid, proposed by Cotta *et al.*^23^Table 3 presents the detailed product distribution with the solvents, along with the initial reaction rate and TOF. It is worth mentioning that the formation of traces of limonene diol (product 8) with DMC can be attributed to the low amount of water present as an impurity in commercial solvents.

### 3.3. Robustness of the dendritic zeolites

**Fig. 8:**
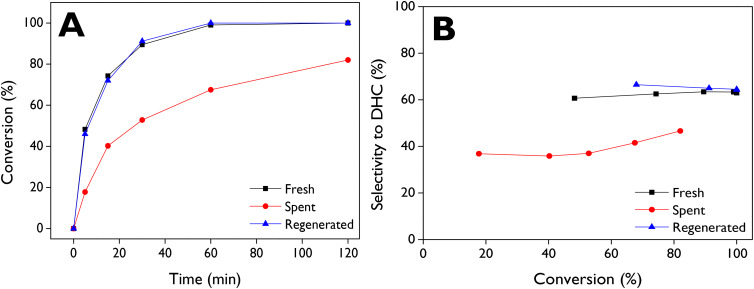
Reusability of d-ZSM-5/4d on the LE isomerization. (A) LE conversion as a function of reaction time and (B) selectivity to dihydrocarvone as a function of the conversion. Reaction conditions: *C*_LE,0_ = 13 mmol L^−1^, 75 mL of total volume, anhydrous ethyl acetate as a solvent, 115 mg of catalyst, 70 °C, 520–530 rpm, N_2_ atmosphere.

The reuse of d-ZSM-5/4d was investigated with two procedures: (i) filtration, washing with acetone, and drying at 100 °C (spent), and (ii) regeneration of the spent catalyst through calcination at 550 °C, with the results shown in Fig. 8. Upon reusing the spent catalyst, a significant loss of activity was observed (Fig. 8A), with about 82% conversion achieved after 2 h, whereas the fresh run exhibited complete conversion after just 1 h. On the other hand, the decrease in selectivity towards DHC (Fig. 8B) with the spent catalyst suggests that the adsorbed species could block preferentially some types of active sites, in particular those responsible for the DHC formation, resulting in a significant change in the product distribution of the isomerization reaction. Conversely, when the spent catalyst was regenerated by air combustion, the catalytic activity was completely recovered, as it is evident in the conversion profile. Moreover, selectivity to DHC was slightly improved compared to the fresh catalyst (Fig. 8B).

TG–air analysis of the spent catalyst sample (Fig. S6†) evidence three main weight losses. The first one, which occurs at low temperatures, is also present in the parent zeolite, being assigned to the removal of physisorbed water. However, two other broad signals at about 280 and 560 °C can be observed in the TG–DTG curves of the spent catalyst, being absent in the case of the parent zeolite. This fact denotes accumulation of some organic matter on the spent catalyst, which is consistent with its light-yellow color. However, these weight losses suppose only 3 wt% (referred to the zeolite weight), which is significantly lower than the value corresponding to the complete filling of the zeolite micropores with organic matter (*ca.* 14 wt%). After regeneration by calcination at 550 °C, a completely white solid was obtained, indicating the total removal of these species. This is confirmed by the TG–DTG profile of the regenerated sample (Fig. S6†), resembling that of the parent zeolite and agreeing with the full recovery of catalytic activity and selectivity towards DHC (Fig. 8).

### 3.4. Extending the scope of dendritic zeolite as catalyst for the isomerization of α- and β-pinene epoxides

The application of dendritic zeolite d-ZSM-5/4d, identified as the most active material in the LE isomerization, was also assessed in the rearrangement of α- and β-pinene epoxides. These epoxides can be synthesized from the well-known corresponding pinenes through the epoxidation route. These catalytic tests were conducted under reaction conditions like those previously described for the LE isomerization, with anhydrous ethyl acetate as the solvent.

The main products obtained from the isomerization of α-pinene epoxide (Fig. S8A†) are campholenic aldehyde (CA), fencholenic aldehyde (FA), *trans*-carveol (TC), pinocamphone (PC), and pinocarveol (PCOL), while *p*-cymene (PCY) can be obtained from dehydration of *trans*-carveol. In the case of β-pinene epoxide isomerization (Fig. S8B†), the main products are myrtanal (*cis* + *trans*), myrtenol, and perillyl alcohol (PA). It is well-known that the production of campholenic aldehyde and myrtanal is favored with catalysts containing Lewis acid sites,^71–73^ as it the case of the dendritic ZSM-5 samples here investigated. Detailed information regarding retention times and mass spectra can be found in the ESI (Tables S4, S5 and Fig. S16–S20†).

**Fig. 9:**
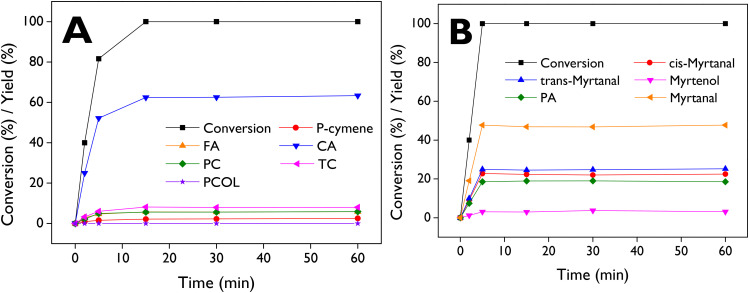
Conversion and product distribution of the isomerization of (A) α-pinene epoxide (60 °C) and (B) β-pinene epoxide (50 °C) over d-ZSM-5/4d. FA: fencholenic aldehyde, CA: campholenic aldehyde, PC: pinocamphone, TC: *trans*-carveol, PCOL: pinocarveol, PA: perillyl alcohol. Reaction conditions: *C*_0_ = 13 mmol L^−1^, 75 mL of total volume, anhydrous ethyl acetate as a solvent, 115 mg of catalyst, 520–530 rpm, N_2_ atmosphere.

Fig. 9A illustrates the complete conversion of α-pinene epoxide after 15 min, with selectivity values of 63%, 8%, 6%, 6%, and 2% for CA, TC, FA, PC, and PCY, respectively. Fig. S9† shows the profiles for the conversion of the epoxide and the yields of the products but using DMC as a solvent at 70 °C, resulting in complete conversion after 5 min and selectivity values of 72% (CA), 5% (TC), 5% (FA), 6% (PC), and 2% (PCY). With β-pinene epoxide (Fig. 9B), complete conversion was reached rapidly after 5 min, with selectivity values of 23%, 25%, 19%, and 3% for *cis*-myrtanal, *trans*-myrtanal, PA, and myrtenol, respectively.

The results demonstrate the promising catalytic activity of d-ZSM-5/4d in the isomerization of pinenes epoxides, leading to complete conversion in very short reaction times and mild reaction temperatures (50–70 °C). Table S2† shows that our results with d-ZSM-5/4d and α-pinene epoxide (entries 1–2) generated higher yields of campholenic aldehyde (62.5% at 60 °C with ethyl acetate after 15 min, and 72.4% at 70 °C with DMC after 5 min) in comparison with entries 3, 5–6, 8–15. Entries 4 and 7 showed slightly higher yields (66% and 64%, respectively) but they were achieved at 70 °C for longer reaction times (2.5 h) and using toluene as the solvent. Moreover, our results with DMC also surpass those two entries. The higher selectivity achieved towards CA with DMC, compared to ethyl acetate, can be easily explained by the lower polarity of DMC (3.09 < 6.02). On the other hand, with β-pinene epoxide as a substrate, d-ZSM-5/4d (entry 16) demonstrated a higher yield of myrtanal (*cis* + *trans*) as the main product than other catalysts described in entries 17–18 and 20–24. A yield to myrtanal of *ca.* 47.5%, like that of d-ZSM-5/4d, was achieved with Sn-Beta-300 (entry 19).^74^ This catalyst exhibits a BA/LA acidity ratio of 0.75, close to the corresponding value of d-ZSM-5/4d (1.4). However, it is noteworthy that Sn-Beta-300 required a higher temperature and longer reaction time. Additionally, a less benign solvent, such as toluene, was used in comparison with ethyl acetate.

## 4. Conclusions

Dendritic ZSM-5 zeolites were tested in the isomerization of monoterpene epoxides such as limonene-1,2-epoxide, α-pinene epoxide, and β-pinene epoxide. The main products of the epoxide rearrangement (dihydrocarvone, campholenic aldehyde, and myrtanal) are high-value compounds used for fragrances, cosmetics, and pharmaceuticals. The study included also conventional and hierarchical ZSM-5 samples as references.

Dendritic ZSM-5 zeolites exhibited a well-crystallized MFI zeolitic structure. The dendritic zeolite with a crystallization time of 4 days (d-ZSM-5/4d) exhibited a remaining mesoscopic ordering, formed during silanization of the synthesis gel, which disappeared when increasing the crystallization time to 7 days (d-ZSM-5/7d). Specifically, the isotherm shape of the dendritic zeolites exhibited a substantial contribution from mesopores, whereas TEM images denote a high degree of connectivity between the different levels of porosities.

The highest TOF (4.4 min^−1^) for limonene-1,2-epoxide (LE) isomerization was achieved with d-ZSM-5/4d zeolite, which exhibited the lowest BA/LA ratio (1.4), the largest mesopore/external surface area (360 m^2^ g^−1^), the narrowest mesopore size distribution and the highest concentration of external Brønsted acid sites (97 μmol g^−1^). This material is significantly more active than catalysts earlier reported based on ordered mesoporous supports such as MCM-41 and SBA-15 with Fe and Cu as active phases. Additionally, this material demonstrated the highest selectivity (60–63%) for *cis*/*trans*-dihydrocarvone (DHC) during the reaction, enabling the DHC yield of 63% after 2 h, thereby surpassing recently reported catalysts for this application. A good correlation was found between the catalytic activity and the concentration of external Brønsted acid sites, showing occurrence of strong steric/diffusional limitations that are mostly overcome when using the dendritic ZSM-5 samples due to their outstanding accessibility.

The reaction rate of LE isomerization is favored in non-polar solvents, while highly selective formation of DHC was observed in the solvents with medium polarity, such as dimethyl carbonate and ethyl acetate. d-ZSM-5/4d demonstrated to be a versatile catalyst for the isomerization also of pinene epoxides, reaching 72% yield of campholenic aldehyde (70 °C, DMC, 5 min) and a 48% yield of myrtanal (50 °C, ethyl acetate, 5 min). These results either surpass or are equivalent to the recent heterogeneous catalysts reported for those routes.

## Author contributions

Luis A. Gallego-Villada: writing – original draft, conceptualization, methodology, investigation, formal analysis. Jennifer Cueto: writing – original draft, investigation. Maria del Mar Alonso-Doncel: writing – original draft, investigation. Päivi Mäki-Arvela: writing – review & editing, conceptualization, supervision. Edwin A. Alarcón: supervision, resources. David P. Serrano: writing – review & editing, conceptualization, resources. Dmitry Yu. Murzin: writing – review & editing, conceptualization, supervision, resources.

## Conflicts of interest

There are no conflicts to declare.

## Supplementary Material

GC-026-D4GC04003A-s001
