# Supplementary material for: Dendritic ZSM-5 zeolites as highly active catalysts for the valorization of monoterpene epoxides
Source: Green Chem. 2024 Sep 16;26(20):10512–28. doi: 10.1039/d4gc04003a (PMC11409433; doi:10.1039/d4gc04003a)
Supplement: GC-026-D4GC04003A-s001 [file GC-026-D4GC04003A-s001.pdf]

Supplementary Information (SI) for Green Chemistry.
This journal is © The Royal Society of Chemistry 2024

SUPPORTING INFORMATION

Dendritic ZSM-5 Zeolites as Highly Active Catalysts for the Valorization of Monoterpene Epoxides

Luis A. Gallego-Villada[a,b,*], Jennifer Cueto[c], María del Mar Alonso-Doncel[c], Päivi Mäki-Arvela[a], Edwin A. Alarcón[b], David P. Serrano[c,d,*], Dmitry Yu. Murzin[a,*]

a. Laboratory of Industrial Chemistry and Reaction Engineering, Johan Gadolin Process Chemistry Centre, Åbo Akademi University, Henriksgatan 2, 20500 Turku/ Åbo, Finland
b. Environmental Catalysis Research Group, Chemical Engineering Faculty, Universidad de Antioquia, Medellín, Colombia
c. Thermochemical Processes Unit, IMDEA Energy Institute, Avda. Ramón de la Sagra, 3, 28935, Móstoles, Madrid, Spain
d. Chemical and Enviromental Engineering Group, Rey Juan Carlos University, c/Tulipán s/n, 28933, Móstoles, Madrid, Spain

* Corresponding author, e-mail address: alfonso.gallego@udea.edu.co (Luis A. Gallego-Villada), david.serrano@imdea.org (David P. Serrano), dmitry.murzin@abo.fi (Dmitry Yu. Murzin)

TABLE OF CONTENT

1. Scheme of the reaction setup.
2. Low-angle XRD patterns.
3. Scheme of reaction products of limonene-1,2-epoxide isomerization.
4. Repeatability test.
5. Confirmation of catalytic route.
6. Turnover Frequency (TOF).
7. TG-Air Analyses.
8. Conversion of limonene-1,2-epoxide isomers.
9. Isomerization of pinene epoxides.
10. Comparison of catalytic systems for the isomerization of pinene epoxides.
11. Characterization of substrate and reaction products.
  11.1. Isomerization of limonene-1,2-epoxide.
  11.2. Isomerization of α-pinene epoxide.
  11.3. Isomerization of β-pinene epoxide.
12. References.

LIST OF TABLES

Table S1. Comparison of turnover of frequency (TOF) for various heterogeneous catalysts in the limonene-1,2-epoxide isomerization.
Table S2. Comparison of catalytic systems for the isomerization of α- and β-pinene epoxides.
Table S3. Retention times for the compounds involved in the isomerization of limonene-1,2-epoxide.
Table S4. Retention times for the compounds involved in the isomerization of α-pinene epoxide.
Table S5. Retention times for the compounds involved in the isomerization of β-pinene epoxide.

LIST OF FIGURES

Figure S1. Reaction setup equipped with a mechanical stirrer, a condenser, a thermocouple, an N_2 feeding, and a sampling valve.
Figure S2. Low-angle XRD patterns of the zeolite samples.
Figure S3. Reaction products of limonene-1,2-epoxide transformations over heterogeneous catalysts based on zeolite ZSM-5.
Figure S4. Repeatability test of d-ZSM-5/4d in the isomerization of LE. **(A)** LE conversion as a function of the reaction time, **(B)** selectivity to dihydrocarvone, **(C)** selectivity to carveol, **(D)** selectivity to product **3**, and **(E)** cis/trans dihydrocarvone mole ratio, as a function of the conversion. **Reaction conditions:** $C_{LE,0}$ = 13 mmol L^{-1}, 75 mL of total volume, anhydrous ethyl acetate as a solvent, 115 mg of catalyst, 70 °C, 520-530 rpm, N_2 atmosphere.
Figure S5. Initial reaction rate of limonene-1,2-epoxide as a function of catalyst mass over d-ZSM-5/7d. **Reaction conditions:** $C_{substrate,0}$ = 13 mmol L^{-1}, 150 mL of total volume, anhydrous ethyl acetate as a solvent, 70 °C, 520-530 rpm, N_2 atmosphere.
Figure S6. TG-DTG (air) analyses of the fresh, spent, and regenerated catalysts of sample d-ZSM-5/4d.
Figure S7. Conversion of cis-limonene-1,2-epoxide (**A, C**) and trans-limonene-1,2-epoxide (**B, D**) as a function of reaction time, evaluating the effect of the catalyst (**A, B**) and the solvent

(**C, D**). Reaction conditions for Figures A and B are described in Figure 5, and those for Figures C and D are described in Figure 7.

Figure S8. Main products of **(A)** α-pinene epoxide and **(B)** β-pinene epoxide rearrangement.

Figure S9. Conversion and product distribution of the isomerization of α-pinene epoxide over d-ZSM-5/4d. FA: Fencholenic aldehyde, CA: Campholenic aldehyde, PC: Pinocamphone, TC: *Trans*-carveol, PCOL: Pinocarveol, PA: Perillyl alcohol. **Reaction conditions:** C_0 = 13 mmol L^{-1}, 75 mL of total volume, dimethyl carbonate as a solvent, 115 mg of catalyst, 70 °C, 520-530 rpm, N_2 atmosphere.

Figure S10. Representative chromatogram of a reaction sample after 2 h in the isomerization of limonene-1,2-epoxide. **Reaction conditions:** $C_{substrate,0}$ = 13 mmol L^{-1}, 75 mL of total volume, DMC as a solvent, 115 mg of d-ZSM-5/4d, 70 °C, 520-530 rpm, N_2 atmosphere.

Figure S11. Mass spectrum of product 3.

Figure S12. Mass spectrum of product 5.

Figure S13. Mass spectrum of product 6.

Figure S14. Mass spectrum of product 7.

Figure S15. Mass spectrum of the main product obtained with acetonitrile as solvent.

Figure S16. Representative chromatogram of a reaction sample after 60 min in the isomerization of α-pinene epoxide. **Reaction conditions:** $C_{substrate,0}$ = 13 mmol L^{-1}, 75 mL of total volume, ethyl acetate as a solvent, 115 mg of d-ZSM-5/4d, 60 °C, 520-530 rpm, N_2 atmosphere.

Figure S17. Representative chromatogram of a reaction sample after 60 min in the isomerization of β-pinene epoxide. **Reaction conditions:** $C_{substrate,0}$ = 13 mmol L^{-1}, 75 mL of total volume, ethyl acetate as a solvent, 115 mg of d-ZSM-5/4d, 50 °C, 520-530 rpm, N_2 atmosphere.

Figure S18. Mass spectrum of product 1.

Figure S19. Mass spectrum of product 2.

Figure S20. Mass spectrum of product 3.

1. Scheme of the reaction setup

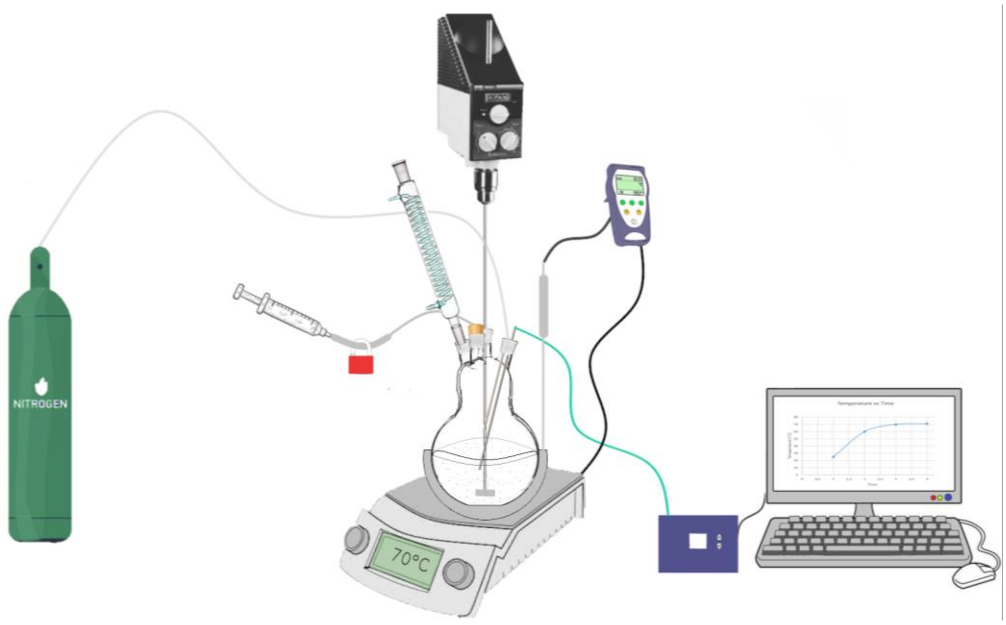

Figure S1. Reaction setup equipped with a mechanical stirrer, a condenser, a thermocouple, an N_2 feeding, and a sampling valve.

2. Low-angle XRD patterns

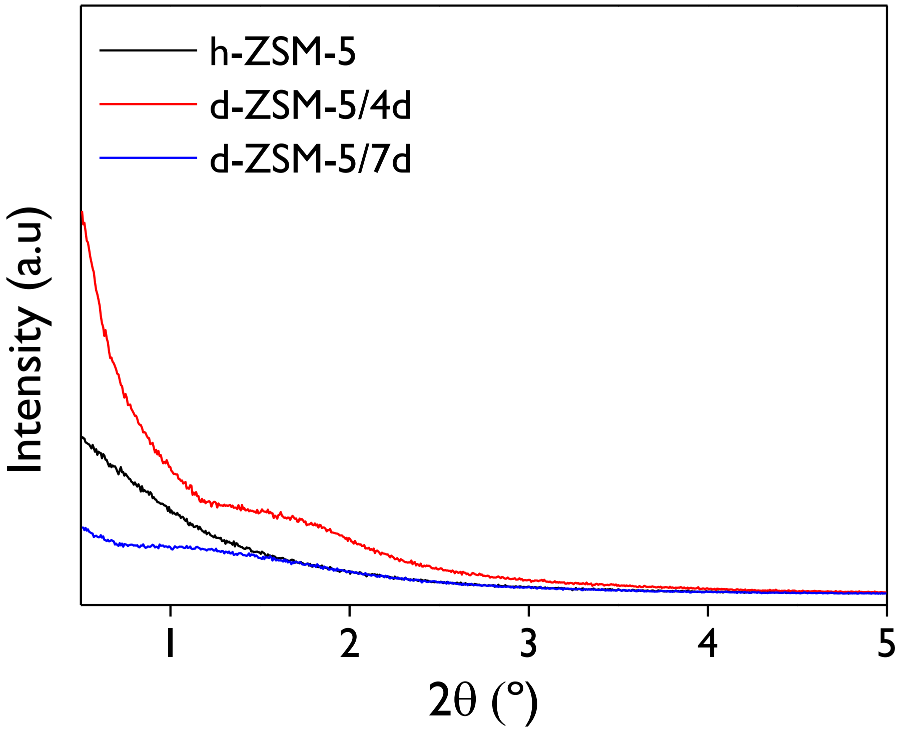

Figure S2. Low-angle XRD patterns of the zeolite samples.

3. Scheme of reaction products of limonene-1,2-epoxide isomerization

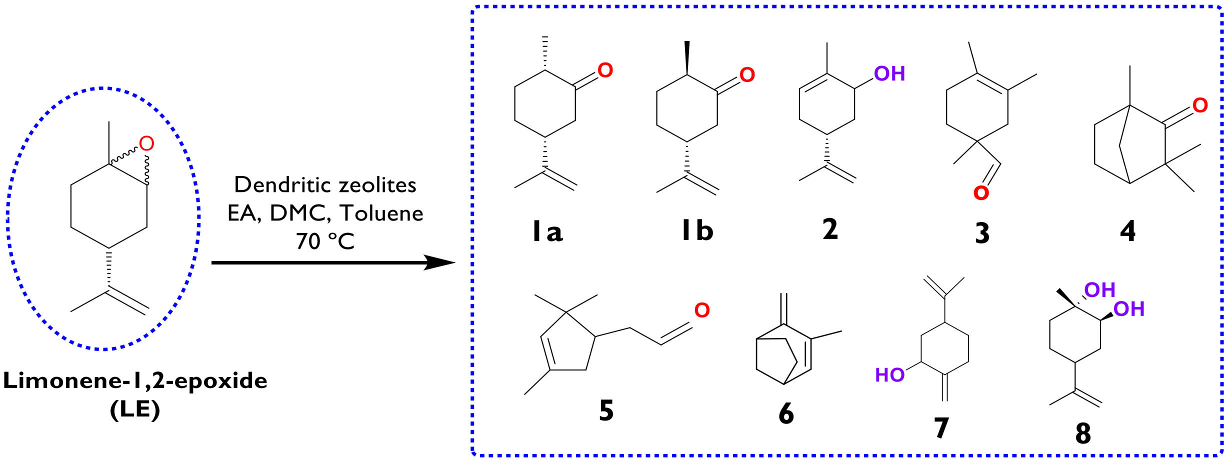

Figure S3. Reaction products of limonene-1,2-epoxide transformations over heterogeneous catalysts based on zeolite ZSM-5.

4. Repeatability test

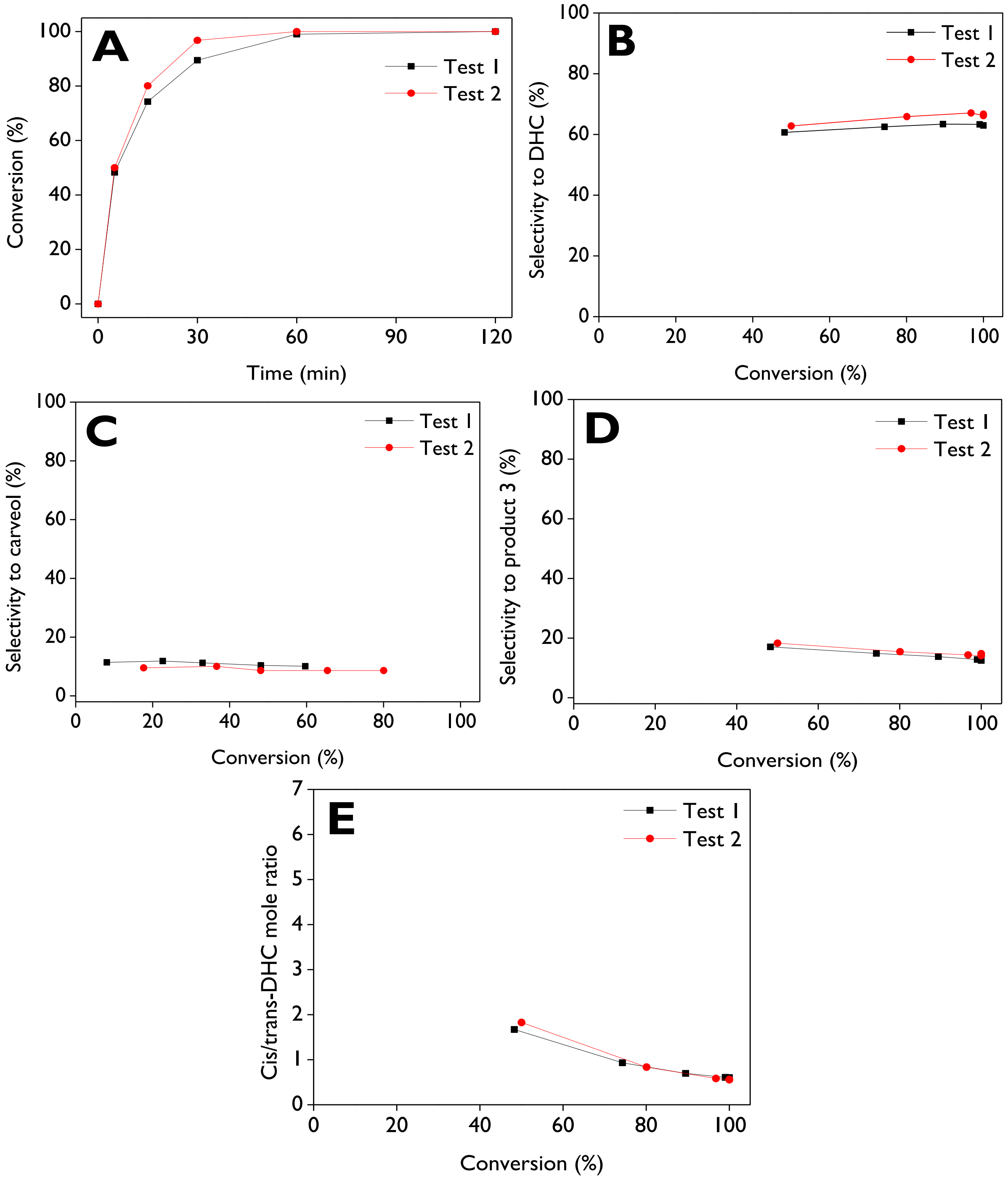

Figure S4. Repeatability test of d-ZSM-5/4d in the isomerization of LE. **(A)** LE conversion as a function of the reaction time, **(B)** selectivity to dihydrocarvone, **(C)** selectivity to carveol, **(D)** selectivity to product **3**, and **(E)** cis/trans dihydrocarvone mole ratio, as a function of the conversion. **Reaction conditions:** $C_{LE,0}$ = 13 mmol L^{-1}, 75 mL of total volume, anhydrous ethyl acetate as a solvent, 115 mg of catalyst, 70 °C, 520-530 rpm, N_2 atmosphere.

5. Confirmation of catalytic route

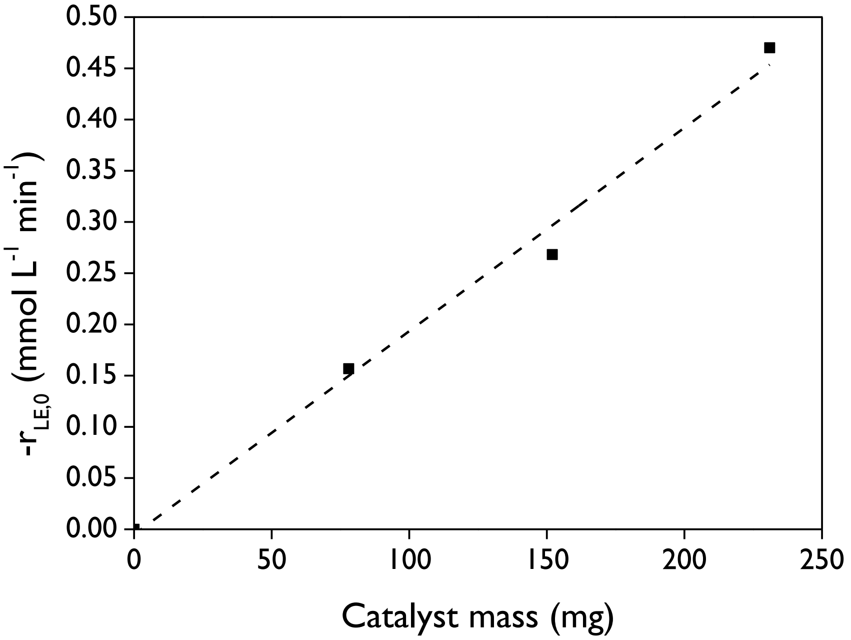

Figure S5. Initial reaction rate of limonene-1,2-epoxide as a function of catalyst mass over d-ZSM-5/7d. **Reaction conditions:** $C_{substrate,0}$ = 13 mmol L^{-1}, 150 mL of total volume, anhydrous ethyl acetate as a solvent, 70 °C, 520-530 rpm, N_2 atmosphere.

6. Turnover Frequency (TOF)

Table S1. Comparison of turnover of frequency (TOF) for various heterogeneous catalysts in the limonene-1,2-epoxide isomerization.

Catalyst	Solvent	Reaction conditions	TOF[b] (min^{-1})	Selectivity to target (%)[d]	Reference
ZSM-5	Ethyl acetate	13 mmol L^{-1}, 75 mL of total volume, 115 mg of catalyst, 70 °C, 520-530 rpm, N_2 atmosphere	0.6	42 (20)	This work
h-ZSM-5	Ethyl acetate		1.6	43 (60)	
d-ZSM-5/4d	Ethyl acetate		4.4	62 (60)	
d-ZSM-5/7d	Ethyl acetate		2.3	55 (60)	
3.9Fe/SBA15[a]	Toluene	0.25 mmol of substrate, 1 mL of solvent, 70 °C, 750 rpm, 10 mg of catalyst	0.46	42 (21)	[1]
	Ethyl acetate		0.29	44 (15)	
	Acetonitrile		0.12	50 (5)	
	Acetone		0.35	0 (17)	
	THF		0.12	48 (5)	
	1,4-Dioxane		0.17	0 (8)	
	Tert-butanol		0.12	13 (5)	
	Cyclohexane		0.23	7 (12)	
	Hexane		0.23	2 (10)	
8.6Fe/MCM41[a,c]	Toluene	0.25 mmol of substrate, 1 mL of solvent, 70 °C, 750 rpm, 25 mg of catalyst	0.8	62 (100)	[1]
	Ethyl acetate		0.8	56 (100)	
	tert-Butanol		0.8	56 (100)	
5.4Cu/MCM4[a,c]	Toluene		0.2	69 (38)	[1]
	Ethyl acetate		0.01	96 (<1)	
	tert-Butanol		0.00	0 (0)	
9.2Fe/SBA15[a,c]	Toluene		0.2	65 (100)	[1]
	Ethyl acetate		0.2	56 (100)	
	tert-Butanol		0.2	50 (100)	
7.2Cu/SBA15[a,c]	Toluene		0.1	67 (41)	[1]
	Ethyl acetate		0.02	80 (6)	
	tert-Butanol		0.04	63 (11)	

[a] The values represent the metal loading (% wt.). [b] TOF was calculated with Eq. (5). [c] Substrate corresponds to α-pinene epoxide. [d] Values in parenthesis indicate the conversion and target corresponds to dihydrocarvone or campholenic aldehyde if the substrate is limonene-1,2-epoxide or α-pinene epoxide, respectively.

7. TG-Air Analyses

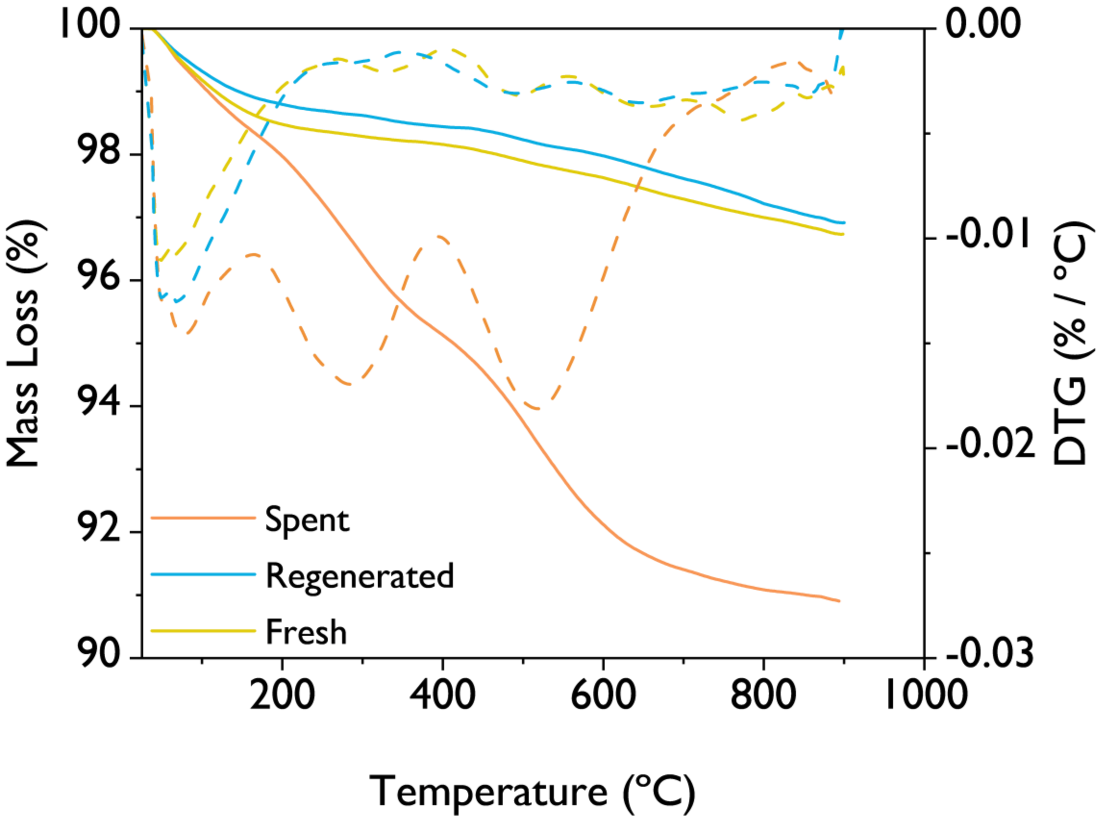

Figure S6. TG-DTG (air) analyses of the fresh, spent, and regenerated catalysts of sample d-ZSM-5/4d.

8. Conversion of limonene-1,2-epoxide isomers

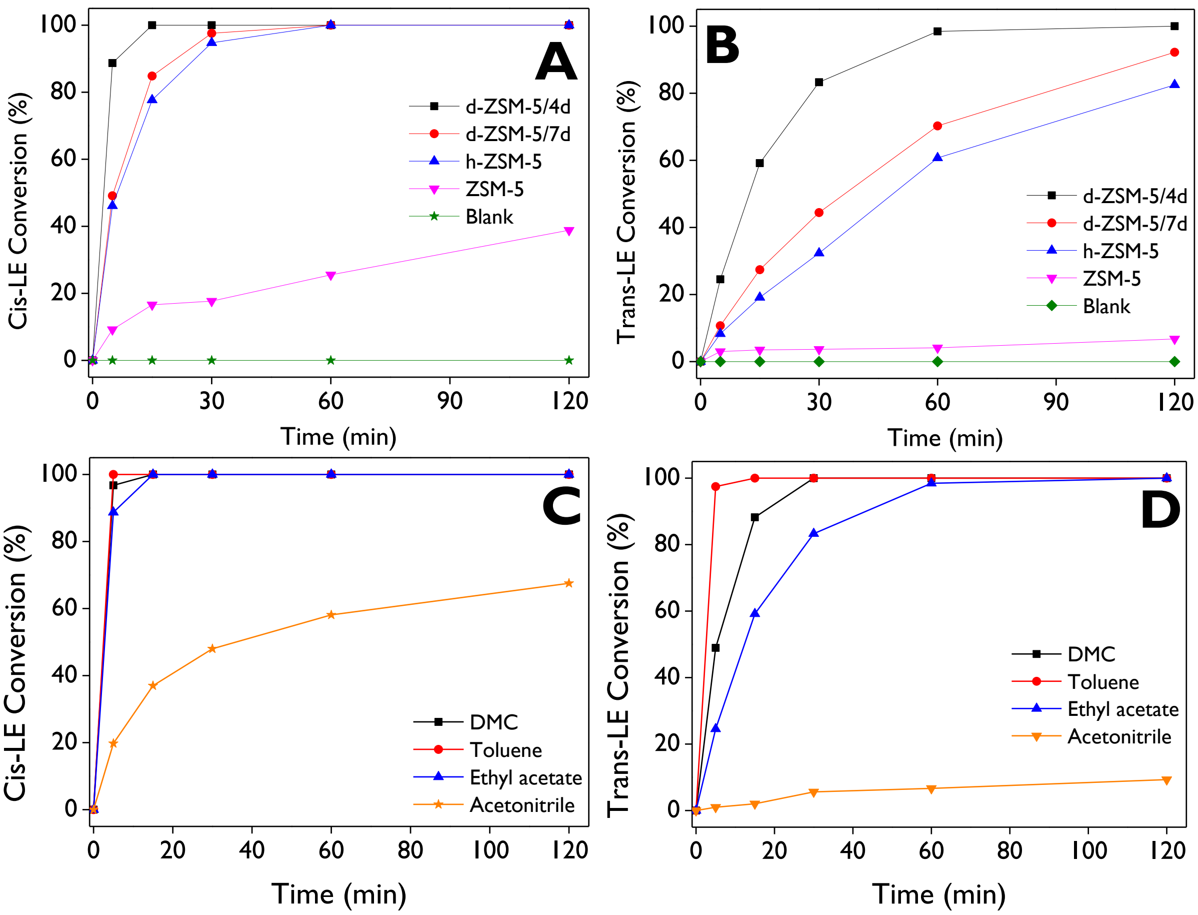

Figure S7. Conversion of cis-limonene-1,2-epoxide (**A, C**) and trans-limonene-1,2-epoxide (**B, D**) as a function of reaction time, evaluating the effect of the catalyst (**A, B**) and the solvent (**C, D**). Reaction conditions for Figures A and B are described in Figure 5, and those for Figures C and D are described in Figure 7.

9. Isomerization of pinene epoxides

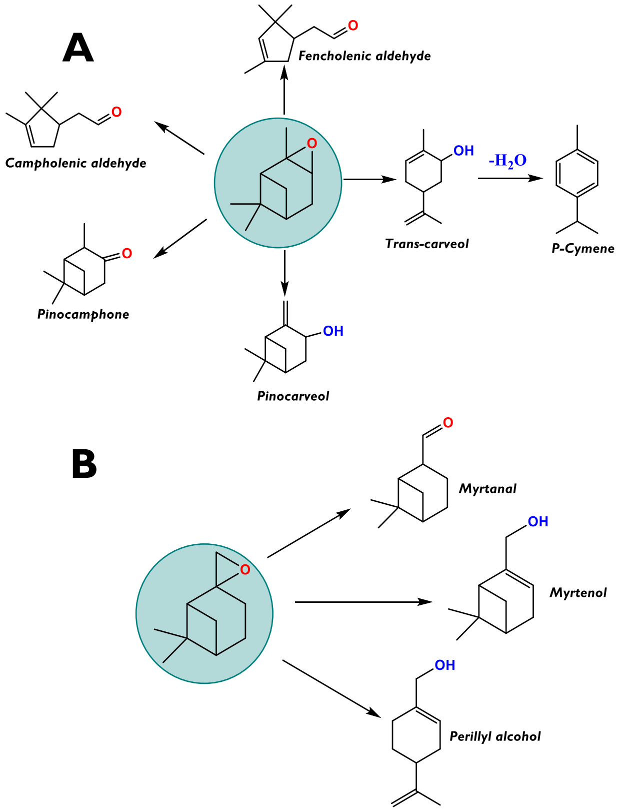

Figure S8. Main products of **(A)** α-pinene epoxide and **(B)** β-pinene epoxide rearrangement.

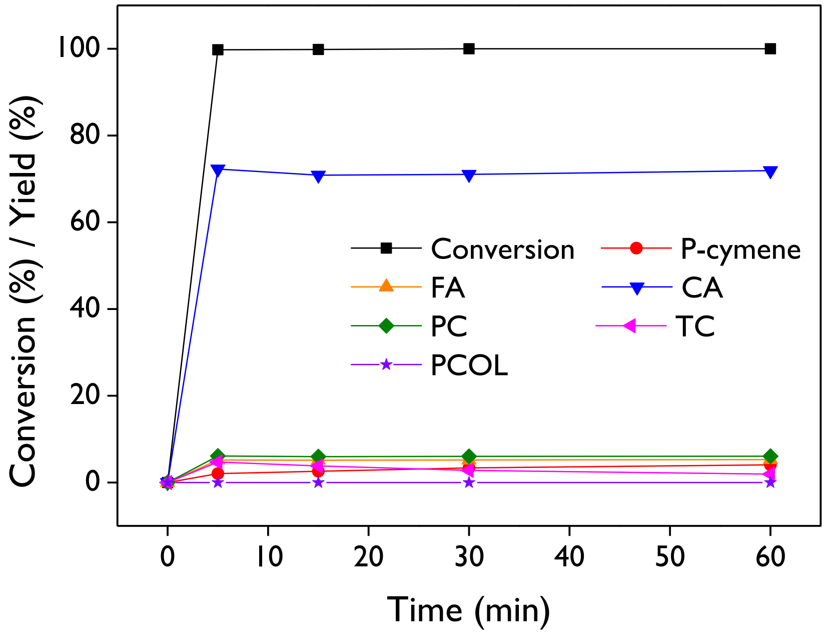

Figure S9. Conversion and product distribution of the isomerization of α-pinene epoxide over d-ZSM-5/4d. FA: Fencholenic aldehyde, CA: Campholenic aldehyde, PC: Pinocamphone, TC: *Trans*-carveol, PCOL: Pinocarveol, PA: Perillyl alcohol. **Reaction conditions:** C_0 = 13 mmol L^{-1}, 75 mL of total volume, dimethyl carbonate as a solvent, 115 mg of catalyst, 70 °C, 520-530 rpm, N_2 atmosphere.

10. Comparison of catalytic systems for the isomerization of pinene epoxides.

Table S2. Comparison of catalytic systems for the isomerization of α- and β-pinene epoxides.

Entry	Substrate	Catalyst	Solvent	Reaction conditions	Conversion (%)	Selectivity (%)	Ref
1	α-Pinene epoxide	d-ZSM-5/4d	Ethyl acetate	13 mmol L^{-1}, 115 mg catalyst, 60 °C, 15 min	100	62.5 CA<br>8.1 TC<br>5.7 FA<br>5.6 PC<br>2 PCY	This work
2	α-Pinene epoxide	d-ZSM-5/4d	Dimethyl carbonate	13 mmol L^{-1}, 115 mg catalyst, 70 °C, 5 min	100	72.4 CA<br>4.7 TC<br>5.2 FA<br>6.1 PC<br>2.1 PCY	This work
3	α-Pinene epoxide	ZrP[a] (2.4 M)	N, N-Dimethylacetamide	3.28 mmol substrate, 50 mg catalyst, 2 mL solvent, 160 °C, 5 h	100	19 CA<br>73 TC	[2]
4	α-Pinene epoxide	Fe/MCM-41 (1.7 wt%)	Toluene	0.25 mmol substrate, 10 mg catalyst, 1 mL solvent, 70 °C, 2.5 h	100	66 CA	[1]
5	α-Pinene epoxide	Fe/MCM-41 (1.7 wt%)	Ethyl acetate	0.25 mmol substrate, 10 mg catalyst, 1 mL solvent, 70 °C, 2.5 h	100	58 CA	[1]
6	α-Pinene epoxide	Cu/MCM-41 (1.3 wt%)	Toluene	0.25 mmol substrate, 10 mg catalyst, 1 mL solvent, 70 °C, 2.5 h	20	82 CA	[1]
7	α-Pinene epoxide	Fe/SBA-15 (3.9 wt%)	Toluene	0.25 mmol substrate, 10 mg catalyst, 1 mL solvent, 70 °C, 2.5 h	100	64 CA	[1]
8	α-Pinene epoxide	Fe/SBA-15 (3.9 wt%)	Ethyl acetate	0.25 mmol substrate, 10 mg catalyst, 1 mL solvent, 70 °C, 2.5 h	100	58 CA	[1]
9	α-Pinene epoxide	Cu/SBA-15 (1.2 wt%)	Toluene	0.25 mmol substrate, 10 mg catalyst, 1 mL solvent, 70 °C, 2.5 h	46	71 CA	[1]
10	α-Pinene epoxide	MZ-5[b] (1.5)	N, N-Dimethylacetamide	2 mmol substrate, 75 mg catalyst, 100 mL solvent, 140 °C, 3 h	92	27 CA<br>45 TC	[3]
11	α-Pinene epoxide	MoO_3-Modified beta zeolite	Toluene	1.25 g substrate, 125 mg catalyst, 6 mL solvent, 70 °C, 3 h	100	34.2 CA<br>14.8 TC<br>14.0 PMD	[4]
12	α-Pinene epoxide	MoO_3-Modified beta zeolite	Ethyl acetate	1.25 g substrate, 125 mg catalyst, 6 mL solvent, 70 °C, 3 h	97	37.1 CA<br>15.8 TC<br>13.9 PMD	[4]
13	α-Pinene epoxide	$Cs_{2.5}H_{0.5}PW_{12}O_{40}$	Acetone	0.75 mmol substrate, 7.50 µmol catalyst, 5 mL total, 40 °C, 5 min	100	17 CA<br>11 TS<br>62 Pinol	[5]
14	α-Pinene epoxide	Al-SiO_2 (12 wt%)	Dichloroethane	0.25 mmol substrate, 5 mg catalyst, 2 mL solvent, 30 °C, 30 min	80	72 CA<br>2 FA<br>15 TC<br>8 TS	[6]
15	α-Pinene epoxide	Phosphonate/Carbon	DMF	3.28 mmol substrate, 50 mg catalyst, 2 mL solvent, 140 °C, 1 h	100	22 CA<br>67 TC<br>9 TPC	[7]
16	β-Pinene epoxide	d-ZSM-5/4d	Ethyl acetate	13 mmol L^{-1}, 115 mg catalyst, 50 °C, 5 min	100	47.7 myrtanal<br>3.1 myrtenol<br>18.5 PA	This work
17	β-Pinene epoxide	Fe/MCM-41	Acetonitrile	0.25 mmol substrate, 26% of catalyst, 1 mL solvent, 70 °C, 1 h	23	90 myrtanal<br>8 PA<br>2 myrtenol	[8]
18	β-Pinene epoxide	Fe/SBA-15	Hexane	0.25 mmol substrate, 26% of catalyst, 1 mL solvent, 70 °C, 1 h	27	68 myrtanal<br>26 PA<br>6 myrtenol	[8]
19	β-Pinene epoxide	Sn-Beta-300	Toluene	0.012 mol L^{-1}, 75 mg of catalyst, 150 mL total, 70 °C, 6 h	72	66 myrtanal<br>10 PA<br>2 myrtenol	[9]

20	Zeolite beta 25	DMSO	0.8 mL substrate, 25 wt% of catalyst (based on the substrate), volume ratio substrate: solvent = 1:5, 70 °C, 2 h	100	19 myrtanal<br>36 PA<br>10 myrtenol<br>9 p-Menth-1-en-7,8-diol	[10]
21	Ti/SBA-15	Hexane	0.25 mmol substrate, 10 mg catalyst, 0.5 mL solvent, 80 °C, 1 h	> 99	20 myrtanal<br>45 PA	[11]
22	Mo/SBA-15	Hexane		> 99	63 PA	
23	Mo/MCM-41	Hexane		98	2 myrtanal<br>20 PA<br>5 myrtenol	
24	Mo/SiO_2	Hexane		99	12 myrtanal<br>32 PA<br>2 myrtenol	

[a]Value in parenthesis refer to the concentration of H_3PO_4 utilized in the synthesis of the catalyst. [b] Selectivities at 70 % conversion. **ZrP:** Zirconium phosphate. **MZ:** Mesoporous beta zeolite where the value in parenthesis denotes the Brønsted-to-Lewis acidity ratio. **CA:** Campholenic aldehyde**. TC:** *Trans*-Carveol. **FA:** Fencholenic aldehyde. **PC**: Pinocamphone. **PCY:** *p*-Cymene. **PMD:** *p*-Methadien-2-ol. **TS:** *trans*-Sobrerol. **TPC:** *Trans*-Pinocarveol. **PA:** Perillyl alcohol.

11. Characterization of substrate and reaction products

11.1. Isomerization of limonene-1,2-epoxide

Table S3. Retention times for the compounds involved in the isomerization of limonene-1,2-epoxide.

Compound	Retention time (min)
Cis limonene-1,2-epoxide	7.17
Trans limonene-1,2-epoxide	7.22
Cis dihydrocarvone (1a)	7.99
Trans dihydrocarvone (1b)	8.10
Carveol (2)	8.32 and 8.49
Product 3	6.81
Fenchone (4)	7.75
Product 5	7.82
Product 6	5.15
Product 7	7.91
Limonene glycol (8)	9.92

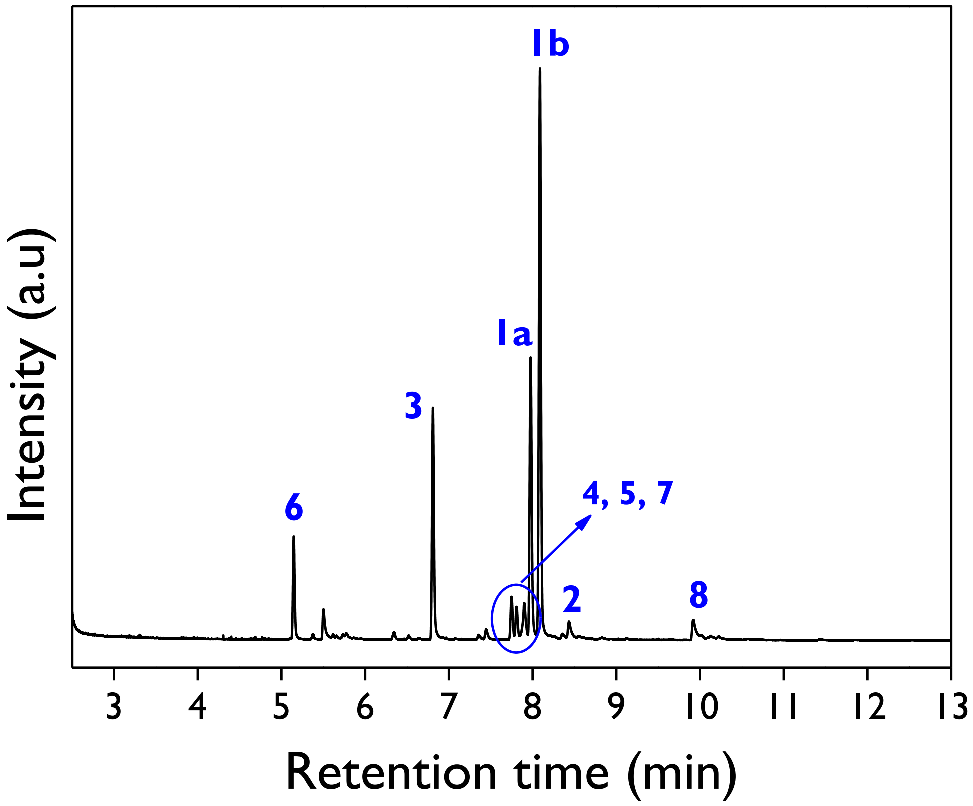

Figure S10. Representative chromatogram of a reaction sample after 2 h in the isomerization of limonene-1,2-epoxide. **Reaction conditions:** $C_{substrate,0}$ = 13 mmol L^{-1}, 75 mL of total volume, DMC as a solvent, 115 mg of d-ZSM-5/4d, 70 °C, 520-530 rpm, N_2 atmosphere.

❖ **Product 3**

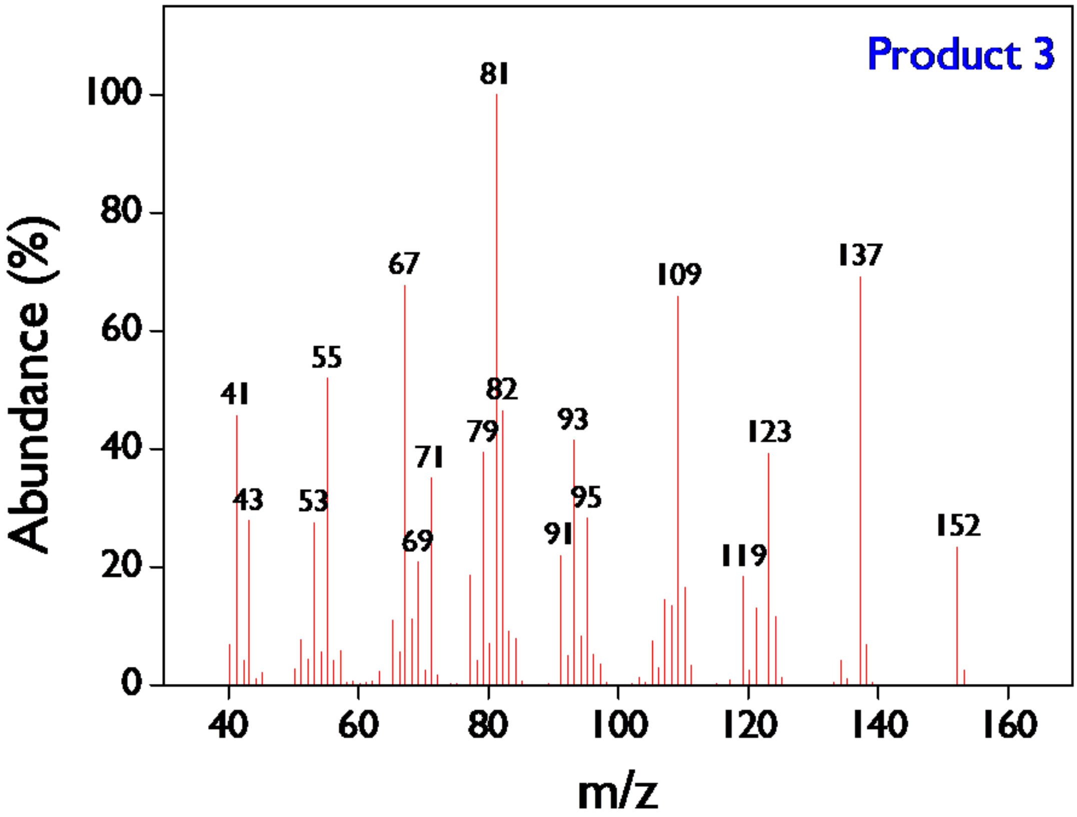

Figure S11. Mass spectrum of product 3.

❖ **Product 5**

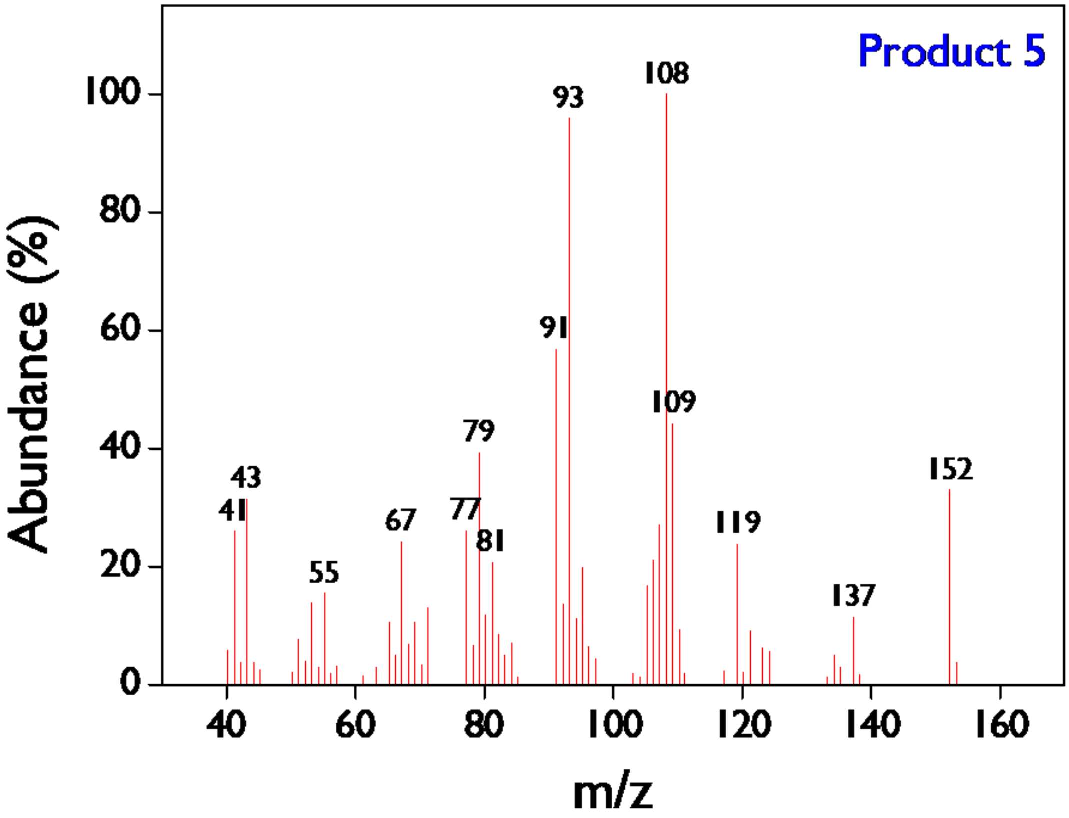

Figure S12. Mass spectrum of product 5.

❖ **Product 6**

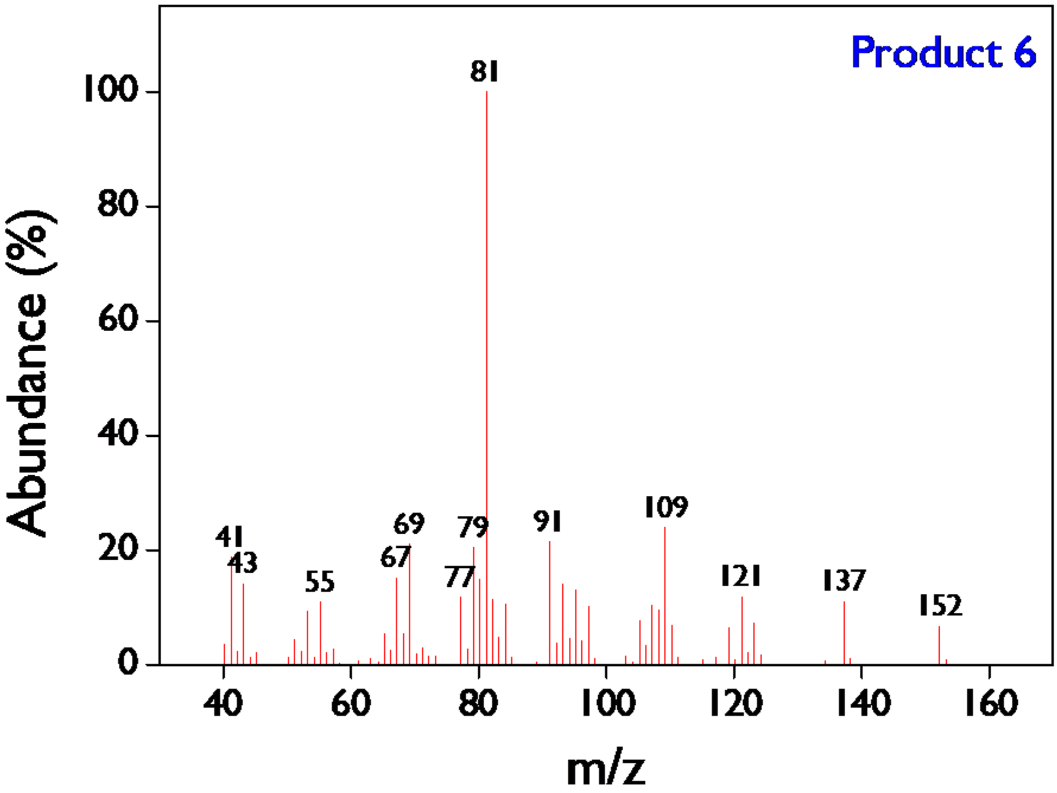

Figure S13. Mass spectrum of product 6.

❖ **Product 7**

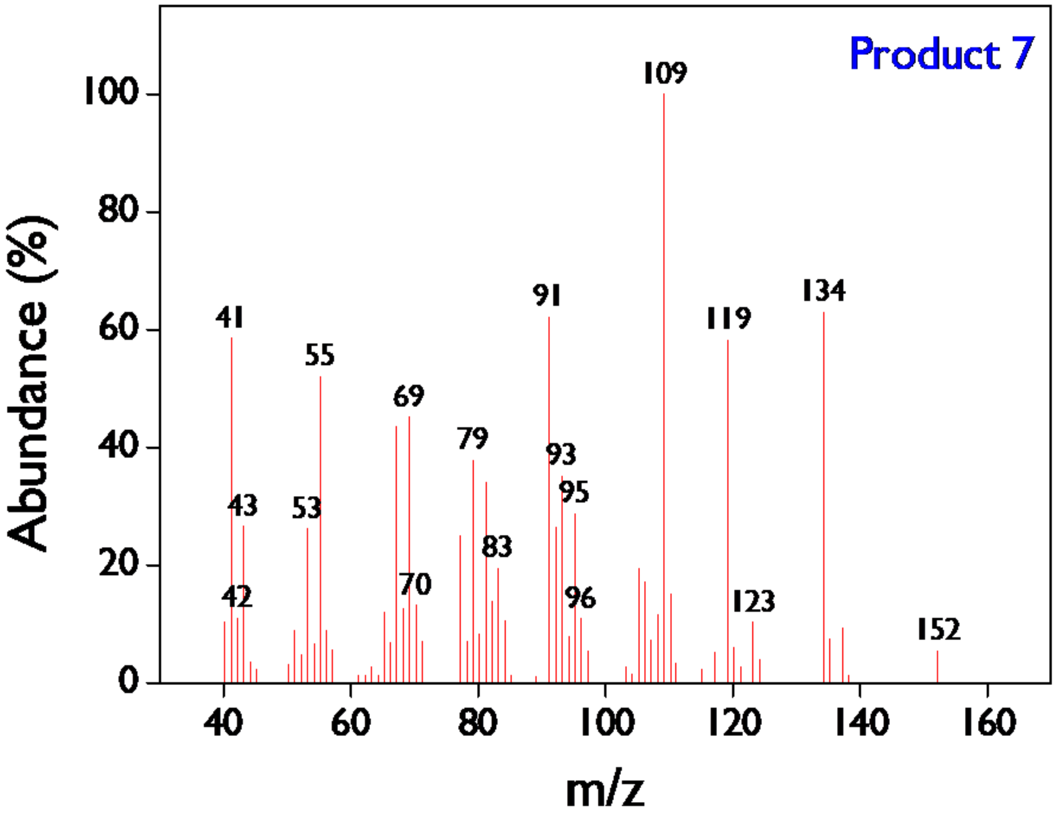

Figure S14. Mass spectrum of product 7.

❖ **Product obtained with acetonitrile as solvent**

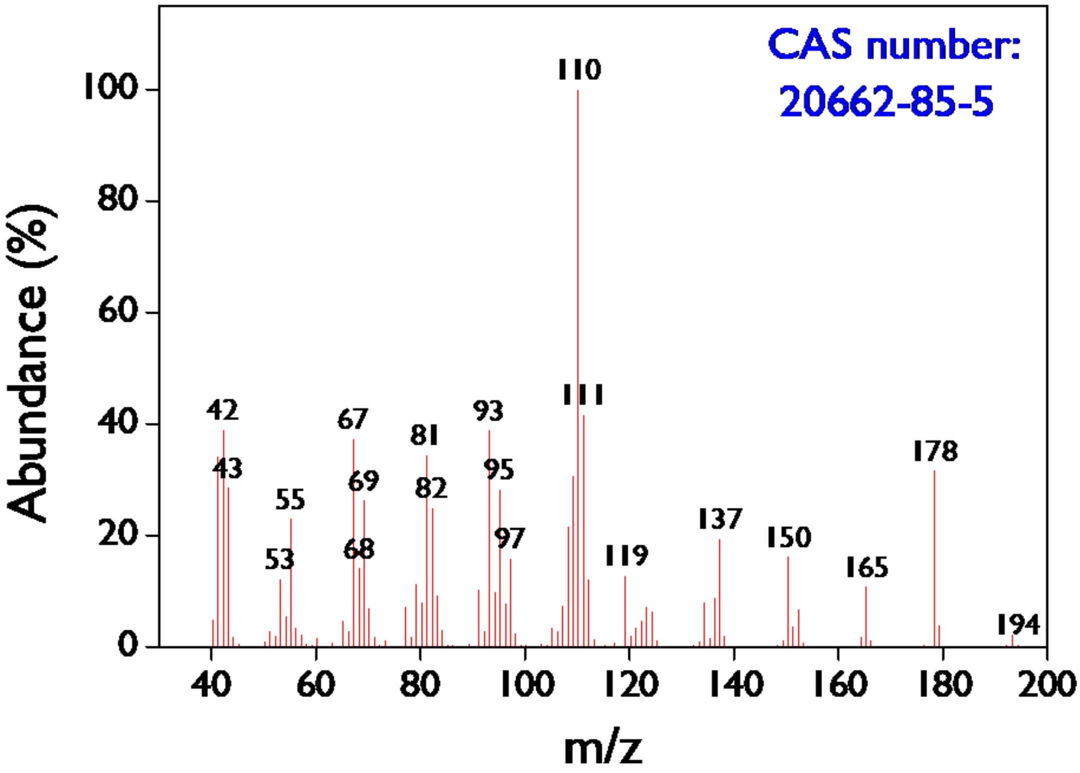

Figure S15. Mass spectrum of the main product obtained with acetonitrile as solvent.

11.2. Isomerization of α-pinene epoxide

Table S4. Retention times for the compounds involved in the isomerization of α-pinene epoxide.

Compound	Retention time (min)
α-Pinene epoxide	6.78
Campholenic aldehyde (1)	7.02
Fencholenic aldehyde (2)	6.47
P-Cymene (3)	5.72
Trans-pinocarveol (4)	7.43
Pinocamphone (5)	7.54
Trans-Carveol (6)	8.32

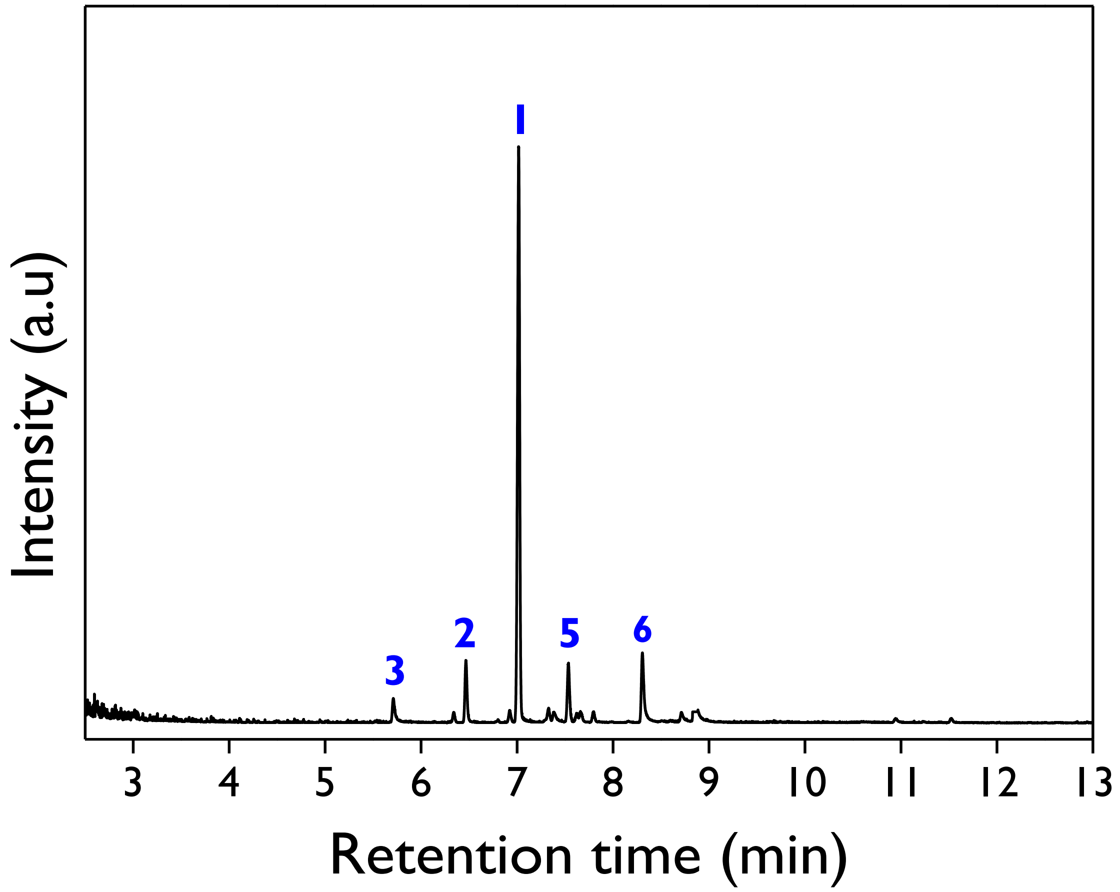

Figure S16. Representative chromatogram of a reaction sample after 60 min in the isomerization of α-pinene epoxide. **Reaction conditions:** $C_{substrate,0}$ = 13 mmol L^{-1}, 75 mL of total volume, ethyl acetate as a solvent, 115 mg of d-ZSM-5/4d, 60 °C, 520-530 rpm, N_2 atmosphere.

11.3. Isomerization of β-pinene epoxide

Table S5. Retention times for the compounds involved in the isomerization of β-pinene epoxide.

Compound	Retention time (min)
β-pinene epoxide	7.55
Product 1 (1)	7.11
Product 2 (2)	7.79
Cis-myrtanal (3a)	7.87
Trans-myrtanal (3b)	7.96
Myrtenol (4)	8.08
Perillyl alcohol (5)	9.45
Product 3 (6)	9.84

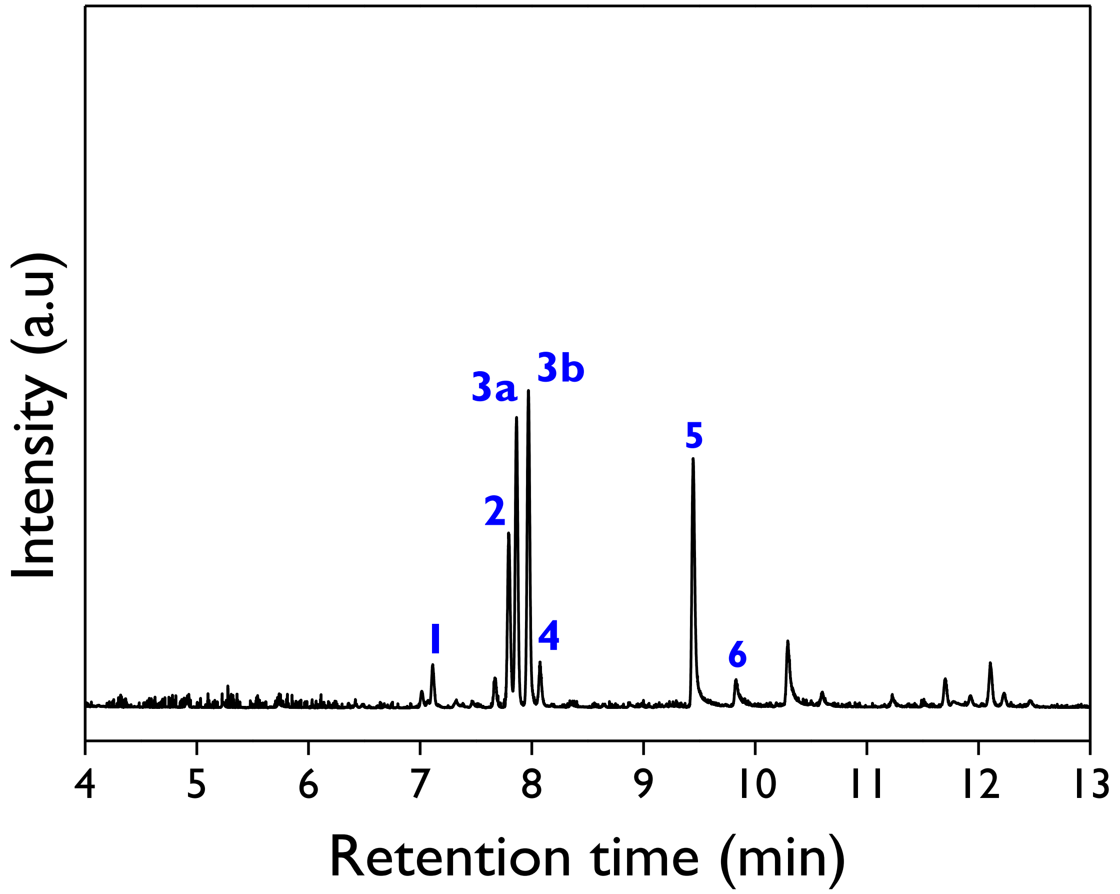

Figure S17. Representative chromatogram of a reaction sample after 60 min in the isomerization of β-pinene epoxide. **Reaction conditions:** $C_{substrate,0}$ = 13 mmol L^{-1}, 75 mL of total volume, ethyl acetate as a solvent, 115 mg of d-ZSM-5/4d, 50 °C, 520-530 rpm, N_2 atmosphere.

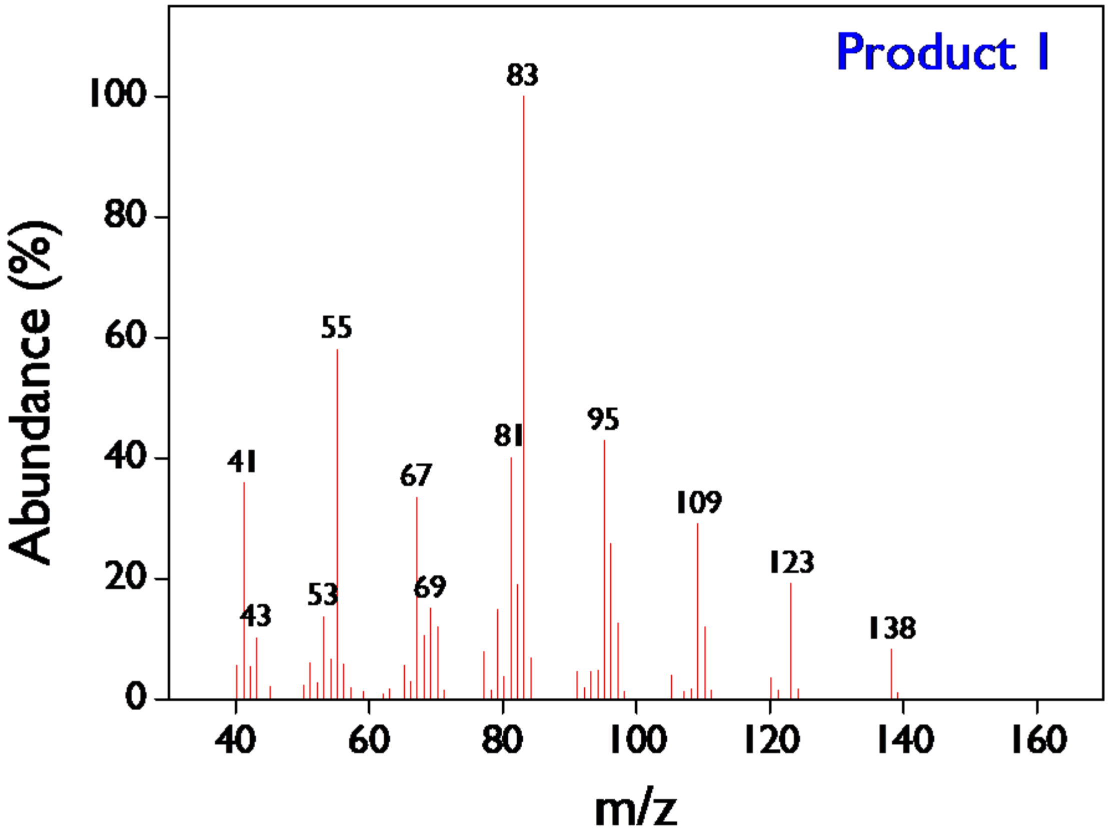

Figure S18. Mass spectrum of product 1.

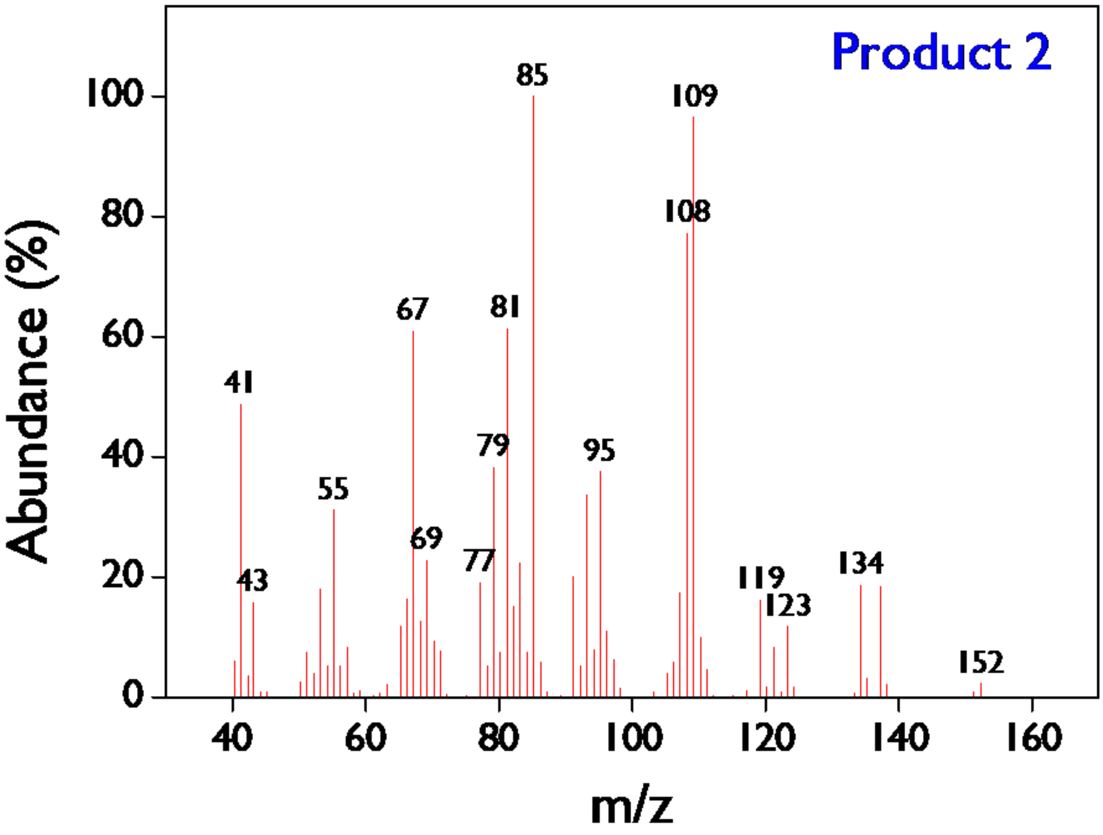

Figure S19. Mass spectrum of product 2.

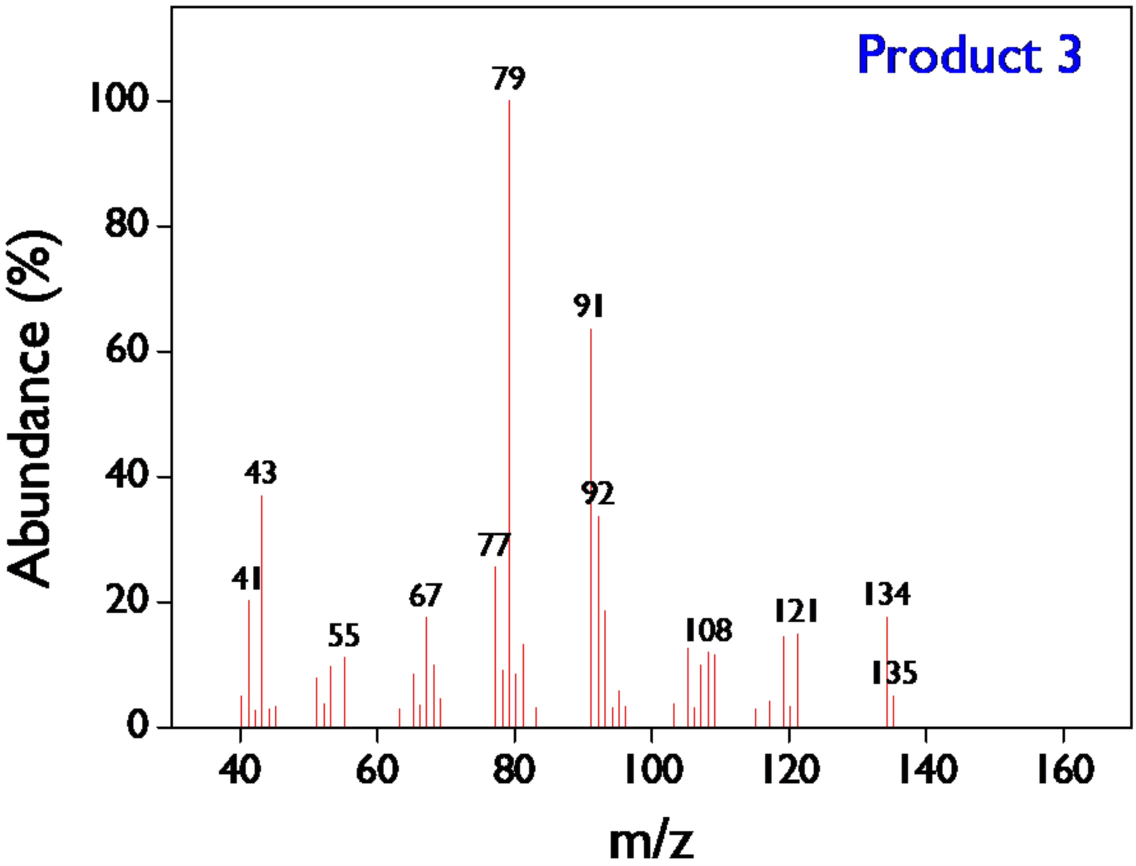

Figure S20. Mass spectrum of product 3.

12. References

[1] J. E. Sánchez-Velandia and A. L. Villa, "Selective synthesis of high-added value chemicals from α-pinene epoxide and limonene epoxide isomerization over mesostructured catalysts: Effect of the metal loading and solvent," *Catal. Today*, vol. 394–396, no. 52, pp. 208–218, Jul. 2022, doi: 10.1016/j.cattod.2021.09.011.

[2] A. S. Singh, D. R. Naikwadi, K. Ravi, and A. V. Biradar, "Chemoselective isomerization of α-Pinene oxide to trans-Carveol by robust and mild Brønsted acidic zirconium phosphate catalyst," *Mol. Catal.*, vol. 521, no. October 2021, p. 112189, Mar. 2022, doi: 10.1016/j.mcat.2022.112189.

[3] R. Barakov *et al.*, "Hierarchical Beta Zeolites As Catalysts in α-Pinene Oxide Isomerization," *ACS Sustain. Chem. Eng.*, vol. 10, no. 20, pp. 6642–6656, May 2022, doi: 10.1021/acssuschemeng.2c00441.

[4] E. Vrbková, E. Vyskočilová, M. Lhotka, and L. Červený, "Solvent Influence on Selectivity in α-Pinene Oxide Isomerization Using MoO3-Modified Zeolite BETA," *Catalysts*, vol. 10, no. 11, p. 1244, Oct. 2020, doi: 10.3390/catal10111244.

[5] C. J. A. Ribeiro, M. M. Pereira, E. F. Kozhevnikova, I. V. Kozhevnikov, E. V. Gusevskaya, and K. A. da Silva Rocha, "Heteropoly acid catalysts in upgrading of biorenewables: Synthesis of para-menthenic fragrance compounds from α-pinene oxide," *Catal. Today*, vol. 344, no. December 2018, pp. 166–170, Mar. 2020, doi: 10.1016/j.cattod.2018.12.023.

[6] V. N. Panchenko, V. L. Kirillov, E. Y. Gerasimov, O. N. Martyanov, and M. N. Timofeeva, "Isomerization of α-pinene oxide to campholenic aldehyde in the presence of Al-SiO2 and magnetic Al-SiO2/Fe3O4 catalysts," *React. Kinet. Mech. Catal.*, vol. 130, no. 2, pp. 919–934, Aug. 2020, doi: 10.1007/s11144-020-01811-x.

[7] A. S. Singh, J. H. Advani, and A. V. Biradar, "Phosphonate functionalized carbon spheres as Brønsted acid catalysts for the valorization of bio-renewable α-pinene oxide to trans -carveol," *Dalt. Trans.*, vol. 49, no. 21, pp. 7210–7217, 2020, doi: 10.1039/D0DT00921K.

[8] M. Chaves-Restrepo, A. Viloria, J. E. Sánchez-Velandia, and A. L. Villa, "Effect of reaction conditions and kinetics of the isomerization of β-pinene epoxide to myrtanal in the presence of Fe/MCM-41 and Fe/SBA-15," *React. Kinet. Mech. Catal.*, vol. 135, no. 4, pp. 2013–2029, Aug. 2022, doi: 10.1007/s11144-022-02220-y.

[9] P. Mäki-Arvela *et al.*, "Isomerization of β-pinene oxide over Sn-modified zeolites," *J. Mol. Catal. A Chem.*, vol. 366, pp. 228–237, Jan. 2013, doi: 10.1016/j.molcata.2012.09.028.

[10] K. Zítová, E. Vyskočilová, and L. Červený, "Preparation of α-terpineol and perillyl alcohol using zeolites beta," *Res. Chem. Intermed.*, vol. 47, no. 10, pp. 4297–4310, Oct. 2021, doi: 10.1007/s11164-021-04515-6.

[11] M. C. Cruz, J. E. Sánchez-Velandia, S. Causíl, and A. L. Villa, "Selective Synthesis of Perillyl Alcohol from β-Pinene Epoxide over Ti and Mo Supported Catalysts," *Catal. Letters*, vol. 151, no. 8, pp. 2279–2290, Aug. 2021, doi: 10.1007/s10562-020-03489-1.
